# Integrating transcriptomics and metabolomics to characterize the regulation of EPA biosynthesis in response to cold stress in seaweed *Bangia fuscopurpurea*

**DOI:** 10.1371/journal.pone.0186986

**Published:** 2017-12-14

**Authors:** Min Cao, Dongmei Wang, Yunxiang Mao, Fanna Kong, Guiqi Bi, Qikun Xing, Zhen Weng

**Affiliations:** 1 Key Laboratory of Marine Genetics and Breeding (OUC), Ministry of Education, Qingdao, P.R. China; 2 College of Marine Life Sciences, Ocean University of China, Qingdao, China; 3 Laboratory for Marine Biology and Biotechnology, Qingdao National Laboratory for Marine Science and Technology, Qingdao, China; Universiti Sains Malaysia, MALAYSIA

## Abstract

*Bangia fuscopurpurea* is a traditional mariculture crop having high nutritional value, eicosapntemacnioc acid (EPA) production, and protein content. As an intertidal species, it can tolerate drastic changes in abiotic factors such as temperature, hydration, and light radiation; however, genomic information on the evolutionary aspect and mechanism of EPA enrichment in *B*. *fuscopurpurea* and the role of EPA in cold tolerance in this species remain elusive. We conducted transcriptome profile analysis in *B*. *fuscopurpurea* to investigate the biological functions of genes associated with resistance to various environment factors. We identified 41,935 unigenes that were assembled and applied to public databases to define their functional annotation (NR, GO, KEGG, KOG, and SwissProt). We further identified genes that encoded key enzymes in EPA biosynthesis; five paralogous genes encoding delta5 desaturase were detected in *B*. *fuscopurpurea*. Fatty acid profiling and gene expression analysis of *B*. *fuscopurpurea* grown under cold stress were simultaneously performed. The EPA content was increased by 29.8% in the samples grown at 4°C, while the total amount of fatty acids remained unchanged. Moreover, all the EPA biosynthesis-related desaturase and elongase genes were upregulated under cold stress. Thus, we hypothesized that diverse EPA biosynthesis pathways and significant increase in gene copy numbers of fatty acid desaturases, together with the concomitant elevation in the transcriptional level of genes associated with fatty acid metabolism, lead to EPA accumulation and subsequently affect membrane fluidity, contributing to cold stress resistance in *B*. *fuscopurpurea*. Our findings not only provide a fundamental genetic background for further research in *B*. *fuscopurpurea*, but also have important implications for screening and genetic engineering of algae and plants for EPA production.

## Introduction

Eicosapentaenoic acid (EPA) is a long-chain ω-3 polyunsaturated fatty acid (PUFA) occurring in various organisms from bacteria to humans as the acyl group of the membrane phospholipids [1]. EPA and its derivatives are key components supporting human health through their anti-inflammatory and cardiovascular health effects [2], which have been attracting widespread attention. Studies have revealed that PUFAs play essential roles in fluidity, acyl chain order, phase behavior, elastic compressibility [3], and permeability of the lipid bilayers but also a beneficial role in membrane organization and cell division [4].

*Bangia fuscopurpurea* is an economically valuable seaweed farmed and harvested in the coastal areas of Zhejiang Province and Fujian Province in China [5]. In China and Southeast Asian countries, *B*. *fuscopurpurea* is a traditional seafood consumed by the vegetarians due to its rich nutrients, such as proteins, free amino acids, polysaccharides, vitamins, and polysaturated fatty acids [6]. EPA content accounts for more than 50% of the total unsaturated fatty acids in *B*. *fuscopurpurea*, which is the highest producer of EPA among the farmed seaweed [7]. However, the lack of genomic information for this species has rendered it difficult to understand the mechanism of high EPA production in this species. *B*. *fuscopurpurea* is also of great interest because of its tolerance to extreme environmental changes in the upper intertidal zone habitat, which is characterized by the ebb and flow of oceanic tides, leading to changes in factors such as temperature, osmotic potential, and light intensity [8]. *B*. *fuscopurpurea* can tolerate temperature changes ranging from 5°C to 30°C during its life history [9]; therefore, it might have developed various effective strategies and mechanisms to overcome the various environmental stresses.

Generally, the fatty acid contents and of an organism varies with the changes of natural environments to maintain the membrane integrity, which represents one of the central mechanisms for abiotic stress tolerance [10]. In plants, a series of sophisticated mechanisms is involved in the response to temperature stress, including fatty acid composition and unsaturation of cells, redistribution of fatty acid species in membrane lipids [11]. In red algal *Porphyridium cruentum*, the direct effect of low temperature on fatty acid composition is an increase in the percentage of 20:4 acids [12]. In green algal *Chlorella minutissima*, the amount of EPA increases by 25% when the culture temperature is changed from 25°C to 20°C [13]. Several tropical microalgae have higher levels of polyunsaturated fatty acids (PUFAs) at low growth temperatures than at high temperatures [14]. The contents of EPA and PUFAs of brown algal *Phaeodactylum tricornutum* were found to be higher at lower temperature [15]. Unfortunately, the roles and the mechanisms of lipids and fatty acids in abiotic-stress tolerance in *B*. *fuscopurpurea* remain unclear. We intended to determine whether the high EPA content in *B*. *fuscopurpurea* is one of the important mechanisms for its temperature stress tolerance. To better understand, the transcriptomic and lipid metabolic profilings under different temperature treatments were generated and the regulation network of fatty acid biosynthesis (especially the EPA synthesis) was deciphered in this study. Those useful genetic and metabolic information will inevitably facilitate a better understanding of the mechanisms of environment adaptation in stress-tolerant macroalgae that grow in intertidal environments and could form a basis for further research.

## Methods

### Material cultivation

*B*. *fuscopurpurea* OUCPT-01 was collected from a farm raft of this species in Nanao Island, Fujian Province. The GPS data of the colleting site is N25°13′38.54″, E119°28′9.87″. The field study was carried out on private farming land which is a cooperative test place of our laboratory. The owner of the land gave permission to conduct this study on this site. Except collected the gametophytes of *B*. *fuscopurpurea*, we had not collected any endangered or protected species. To remove sand and attached epiphytes (diatoms, blue algae, etc.), we first brushed *B*. *fuscopurpurea* and rinsed them several times with sterilized seawater. Next, we selected a clean gametophyte and cultured it in Provasoli’s enrichment medium prepared using sterilized seawater [16] at 20 ± 1°C under a photon flux density of 60 μmol photons·m^−2^·s^−1^ with a 12 h:12 h light-dark cycle. The archeospores released by this gametophyte developed into gametophytes after 7 days. The offspring were then transferred into another culture medium. After three generations of inoculation, *B*. *fuscopurpurea* were mass cultured for the following experiments. The stock of *B*. *fuscopurpurea* was maintained in the germplasm bank at the Ocean University of China. The *B*. *fuscopurpurea* gametophyte had uniseriate or multiseriate unbranched filaments. Each cell in the gametophyte contained a massive stellate chloroplast with a single pyrenoid (S1 Fig).

### Collecting multiple stress-treated samples for transcriptome sequencing

To maximize the coverage of transcriptome sequencing, we collected the gametophytes subjected to various abiotic stress conditions, including different temperature (4°C, 10°C, 20°C, and 30°C), light (0 μmol and 400–500 μmol photons·m^−2^·s^−1^), osmotic (materials with water content of 30% and 70% and rehydration for 40 min), salinity (0‰, 17‰, and 66‰), rhythm (materials were collected before 1 and after 3 h of the light cycle and before 1 h of the dark cycle), and non-nutrient (cultured under lack of nutrition for 5 days) conditions. The conditions are described in detail in additional S1 Table. Three biological replicates were used for normal condition and for each stressed condition. For each stress-treated *B*. *fuscopurpurea* sample, total RNA was isolated using an RNeasy® Plant Mini kit (Cat. No. 74904; Qiagen) following manufacturer’s instructions, and then treated with RNAse-free DNase I (Omega Bio-Tek, Doraville, USA) to remove genomic DNA contamination. The purity and concentration of the RNA were detected using NanoPhotometer^®^ spectrophotometer (IMPLEN, CA, USA), and the integrity was determined using an Agilent 2100 Bioanalyzer (Agilent, Santa Clara, CA).

### Transcriptome data sequencing and *de novo* assembly

About 30 μg RNA from the stress-treated samples and normal-cultured samples was mixed together and used to construct the transcriptome sequencing library. The mRNA was first purified from the total RNA by using Dynabeads® Oligo (dT)_25_ (Invitrogen 610–05, USA) and then fragmented. Double-stranded cDNA was synthesized using transcriptase and random hexamer primers. The sequencing library was constructed using the NEBNext^®^ UltraTM RNA Library Prep Kit (NEB, USA) and then loaded onto an Illumina HiSeq2000 platform for PE 2 × 100 bp sequencing. In all, 35,534,655 paired-end reads were obtained and used for the following analysis. The generated raw sequencing data was available in the Sequence Read Archive (BioProject ID: PRJNA404009), NCBI.

Raw reads were first subjected to preliminary processing by using the FASTX-Toolkit (http://hannonlab.cshl.edu/fastx_toolkit/) to remove reads that contained adapter sequences, poly-N sequences (greater than 10%), low-quality reads, and reads with lengths less than 40 bp. The clean reads were assembled using Trinity v2013-02-25 by using the default parameters (http://trinityrnaseq.sourceforge.net/). Redundant transcripts were avoided by extracting the longest transcript of each gene as a unigene for downstream analysis.

### Functional annotation of *B*. *fuscopurpurea* unigenes

We used the “GetORF” program in the EMBOSS toolkit [17] to predict the open reading frames (ORFs) for the unigenes. These ORFs were then searched against the non-redundant protein (NR) database by using the Blastp program with an E-value cutoff of 1e-5 for functional annotation. Gene Ontology (GO) annotation was performed using Blast2GO program [18], and metabolic pathways were assigned by blasting these sequences against the Kyoto Encyclopedia of Genes and Genomes (KEGG) database by using Blastx. Annotation in KOG and SwissProt databases was also performed.

### Identification of genes involved in EPA biosynthesis and phylogenetic analysis

Protein sequences of genes involved in fatty acid and EPA biosynthesis in model organisms and phylogenetically close species were collected from GenBank and are listed in S2 Table. They were used as queries to blast against the unigenes by using the default parameters. A unigene hit meeting the following two criteria was inferred to be homolog: (a) it was functionally annotated in NR and KEGG databases, and (b) it was grouped with homologous genes from model organisms in phylogenetic trees. For fatty acid desaturases, protein sequences from *B*. *fuscopurpurea* and other model organisms were first aligned using the CLUSTAL X v. 1.81 program [19]. Both ML and Bayesian trees were constructed using RAxML and MrBayes3.2 [20–21], respectively. The optimal evolutionary model for the dataset was determined using the Model test [22].

### Collection of temperature-stressed samples and gene expression analysis

For determining the changes in gene expression of *B*. *fuscopurpurea* subjected to cold stress, we cultured the gametophytes of *B*. *fuscopurpurea* under normal temperature (20°C) and under cold stress by directly placing the samples in pre-cooled seawater at 4°C and 10°C for 30 min. Each treatment was in performed in triplicate; total RNA was then extracted. The isolated total RNAs were quantified using Qubit 2.0. The integrity of RNA was detected using Agilent 2100 (S3 Table). We used an equal amount of RNA from each sample for reverse transcription as described above.

For all the EPA biosynthesis-related desaturase and elongase genes (*delta4*, *delta5*, *delta6*, *delta9*, *delta12*, *Elo2*, *Elovl2*, *FabI*, and *FabH*), one gene was selected for examination at the transcriptional level under different stress conditions by using qRT-PCR. Primers were designed to amplify a 100–200-bp fragment in the coding region for each target gene. All the primer sequences for target and reference genes are listed in S4 Table. The amplified fragments were first cloned into a pMD18T (TaKaRa Code: D103A) vector, and the cloned fragments were then diluted by 10 folds and used as PCR templates for calculating a standard curve for the qRT-PCR results. Dissociation curves were also analyzed for each primer to detect nonspecific amplification and primer dimer production. The efficiency (E) of the reaction for each primer was calculated using the formula E = (10^−1/slope^-1). The expression levels of these genes were analyzed using qRT-PCR by using a Light-Cycle® 480 Real-Time PCR System (Roche Applied Science). The reactions were performed in 20 μL volumes that contained 10 μL Light Cycler 480 SYBR Green I Master (Roche Applied Science), 0.6 μL each primer, 2 μL diluted cDNA, and 6.8 μL RNA-free water. For the qRT-PCR, 45 cycles were run as follows: 95°C for 10 s, 57°C for 10 s, and 72°C for 20 s. The *tublin* and *GAPDH* genes were used as internal transcription controls. The 2^-ΔΔ^Ct method was used to calculate the relative gene expression values [23]. All the raw data are on cycle thresholds, and the statistical analysis results are shown in S5 Table.

### Analysis of fatty acids

Triplicate samples of lyophilized *B*. *fuscopurpurea* gametophytes from normal control (20°C) and cold stress conditions (4°C, 10°C) were analyzed for total lipid and fatty acid composition. Total lipids were extracted using the method described by Bligh & Dyer [24], and then the fatty acid methyl esters were prepared using transesterification of the lipid extracts by incubation in 15% BF_3_-methanol at 80°C for 90 min [25]. After transesterification, the generated methyl esters were loaded onto a QP2010 GC-MS (Shimadzu, Japan) for fatty acid analysis in an SPB-50 fused silica capillary column (30 m × 0.25 mm× 0.25 mm).

## Results

### Sequencing and *de novo* assembly of the *B*. *fuscopurpurea* genome

**Fig 1:**
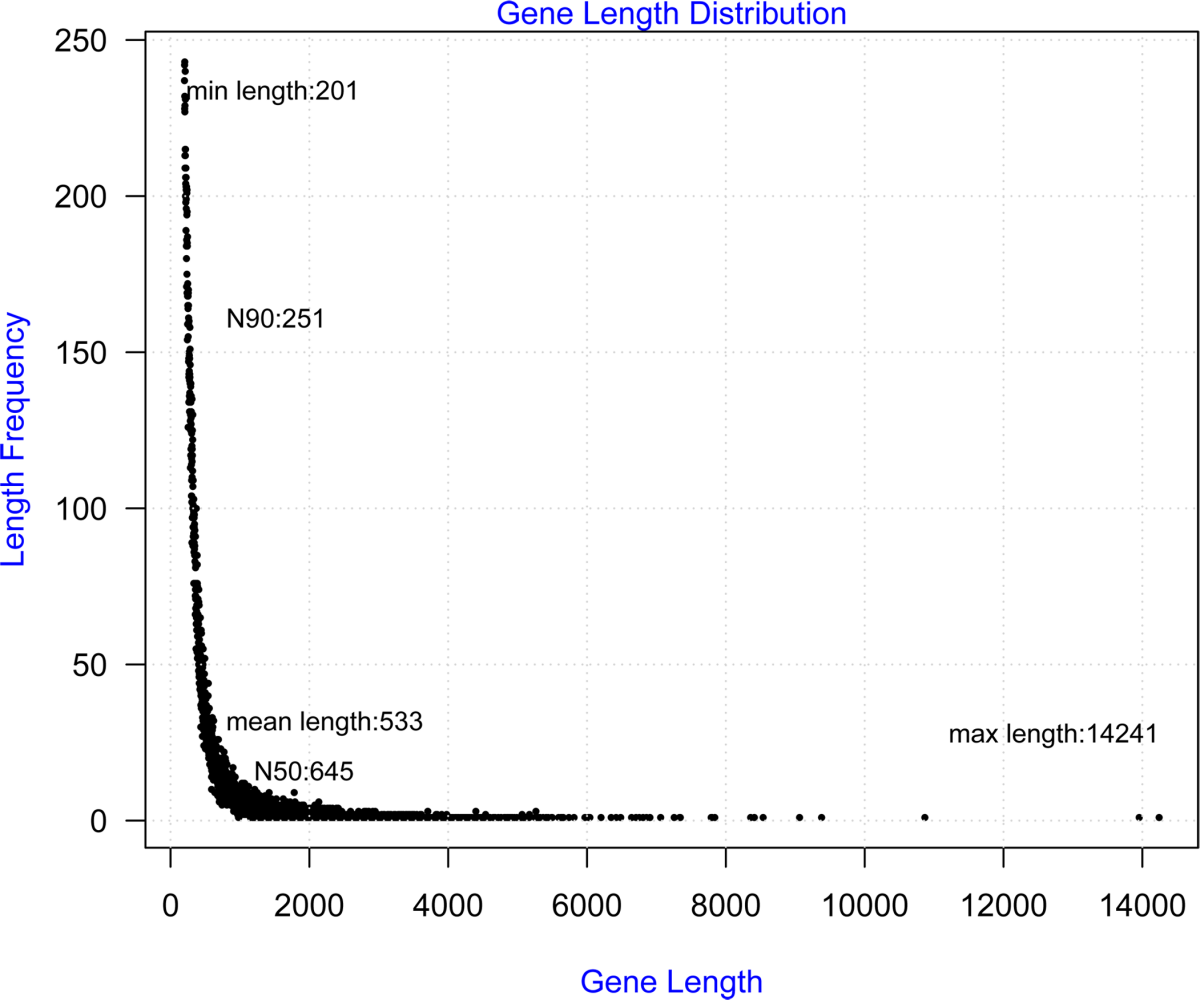
The length distribution of unigenes identified in *Bangia fuscopurpurea*.

To characterize the functional gene sets encoded in the *B*. *fuscopurpurea* genome, especially those involved in stress response and adaptation, we collected *B*. *fuscopurpurea* gametophytes grown under both normal and various stress conditions (S1 Table) to isolate total RNA for Illumina mRNA-seq analysis. We generated a total of 35,534,665 paired-end reads 100 bp in length. After low-quality reads and those with adapter sequences were removed, 31,173,496 (87.7%) clean reads with 63.99% GC content were obtained. Clean reads were assembled *de novo* into 41,935 unigenes on the Trinity platform (Table 1). The length of these assembled unigenes ranged from 201 to 14,241 bp, with the average length of 533 bp and an N50 of 645 bp. We found that 28.3% of the unigenes were longer than 500 bp, and 10.5% were longer than 1,000 bp (Fig 1).

**Table 1:** Summary of the *Bangia fuscopurpurea* transcriptome.

Item	Number
Total number of raw reads	35,534,665
Total number of clean reads	31,173,496
Total base pairs (bp)	6,234,699,200
Average read length	100 bp
Q20	97.46%
GC percentage	63.99%
Total number of unigenes	41,935
Average length of unigenes	533
Min length	201
Max length	14,241
N50 length	645

### Functional annotation and classification of the assembled unigenes

To investigate the biological functions of the assembled unigenes, we first mapped them onto the NCBI NR database and the SwissProt protein database by using the BLASTx algorithm with an E-value threshold of 1e-5. We identified 8,134 (19.4%), 5,688 (13.6%), 7,227 (17.2%), and 8,708 (20.8%) of the 41,935 unigenes that had at least one hit in NR, SwissProt, TrEMBL, and Uniprot databases, respectively (S6 Table). These annotated unigenes formed a potential pool for gene identification in *B*. *fuscopurpurea*. Of the genomes that harbored the best Blast hits for *B*. *fuscopurpurea* unigenes in the NR database, the top ten species were *Chondrus crispus*, *Acanthamoeba castellanii*, *Galdieria sulphuraria*, *Ectocarpus siliculosus*, *Polysphondylium pallidum*, *Saprolegnia diclina*, *Guillardia theta*, *Dictyostelium discoideum*, and *Dictyostelium fasciculatum*.

**Fig 2:**
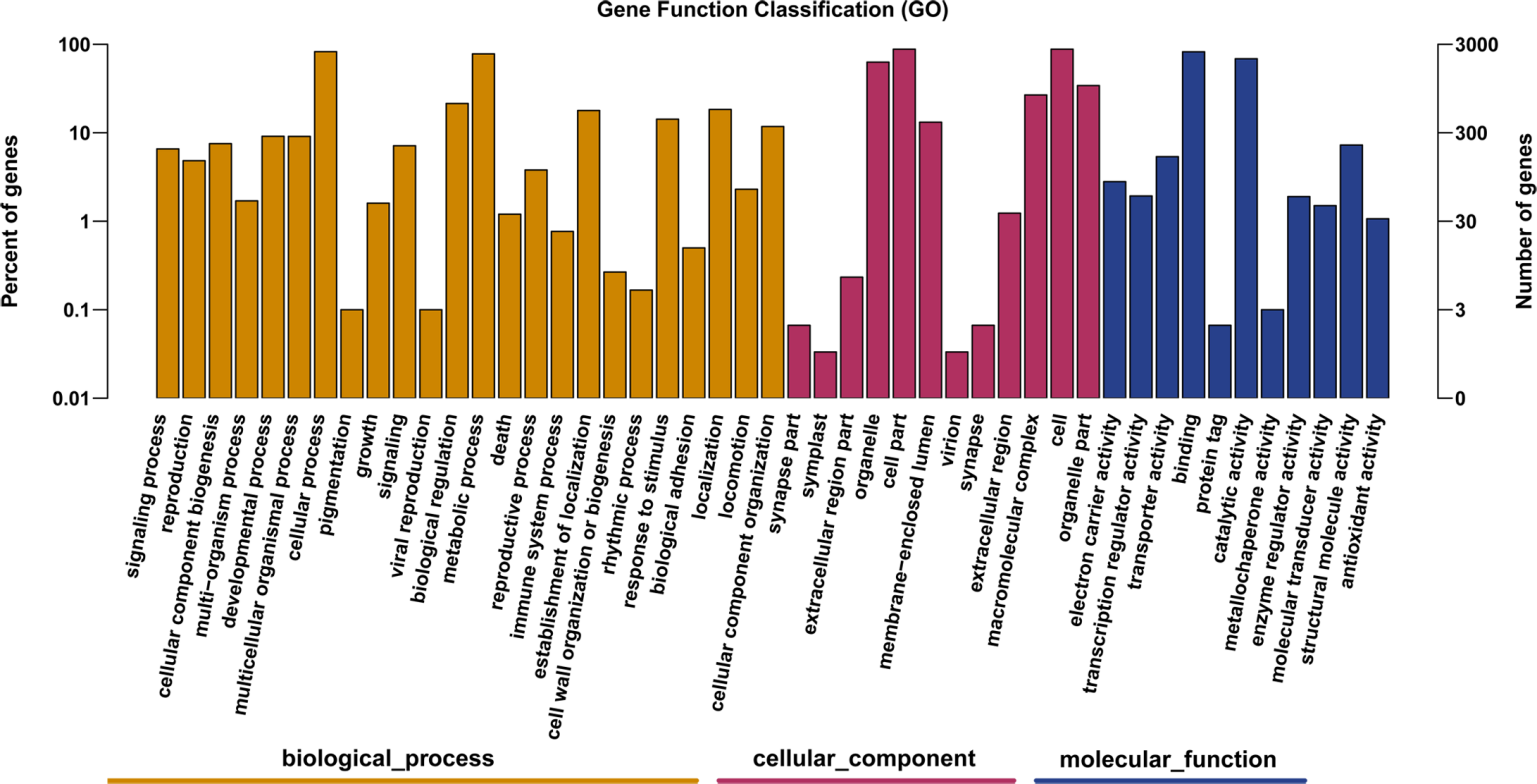
Functional classification of *Bangia fuscopurpurea* unigenes by using Gene Ontology (GO) terms.

A GO analysis was conducted with the *B*. *fuscopurpurea* unigenes by using the Blast2GO program to classify their biological functions. In all, 3,164 unigenes were successfully assigned to GO glossaries with 23,645 functional terms (Fig 2). Among them, 8,596 (38.2%), 5,184 (21.9%), and 9,438 (39.9%) unigenes were, respectively, assigned in the following categories: biological process, molecular function, and cellular component. In the biological process category, the most abundant functional groups in *Bangia* were metabolic and cellular processes, biological regulation, cellular localization, and response to stimulus. In the molecular function category, catalytic activity, binding, structure molecule activity, and transporters were the most highly represented groups. In the cellular component category, cell, cellular parts, organelles, and macromolecular complex were the four major groups associated with the *B*. *fuscopurpurea* unigenes.

**Fig 3:**
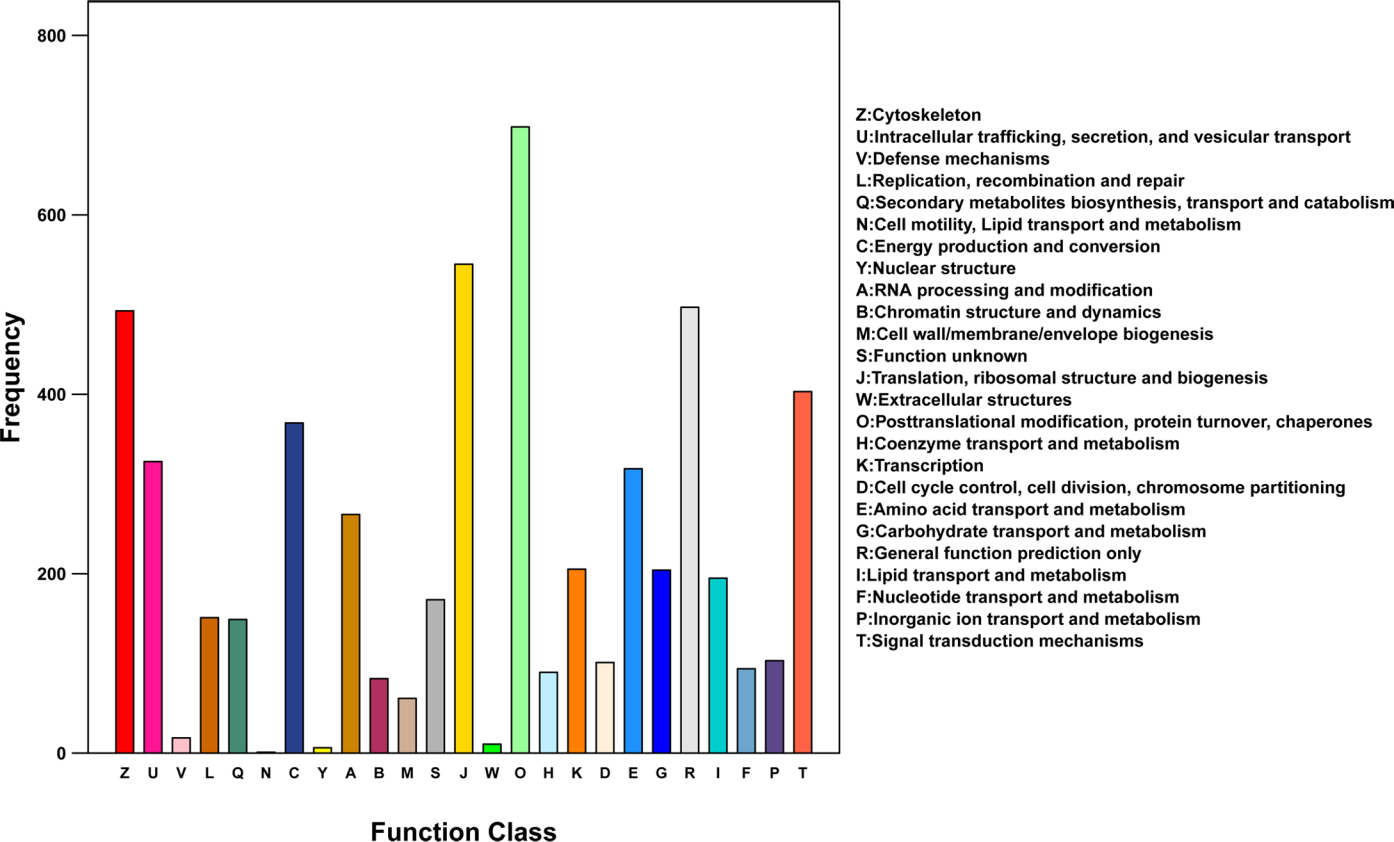
The orthologous groups (COG) encoded by unigenes in the *Bangia fuscopurpurea* transcriptome.

All of the annotated unigenes were aligned to the KOG database to predict and classify their possible functions. A total of 5,552 sequences were assigned to the KOG classifications (Fig 3). Among the 25 KOG categories, the clusters for posttranslational modification, protein turnover, and chaperones (698, 12.6%) represented the largest groups, followed by translation, ribosomal structure, and biogenesis (545, 9.8%); general function prediction only (497, 9.0%); cytoskeleton (493, 8.9%); and signal transduction mechanisms (404, 7.3%).

**Fig 4:**
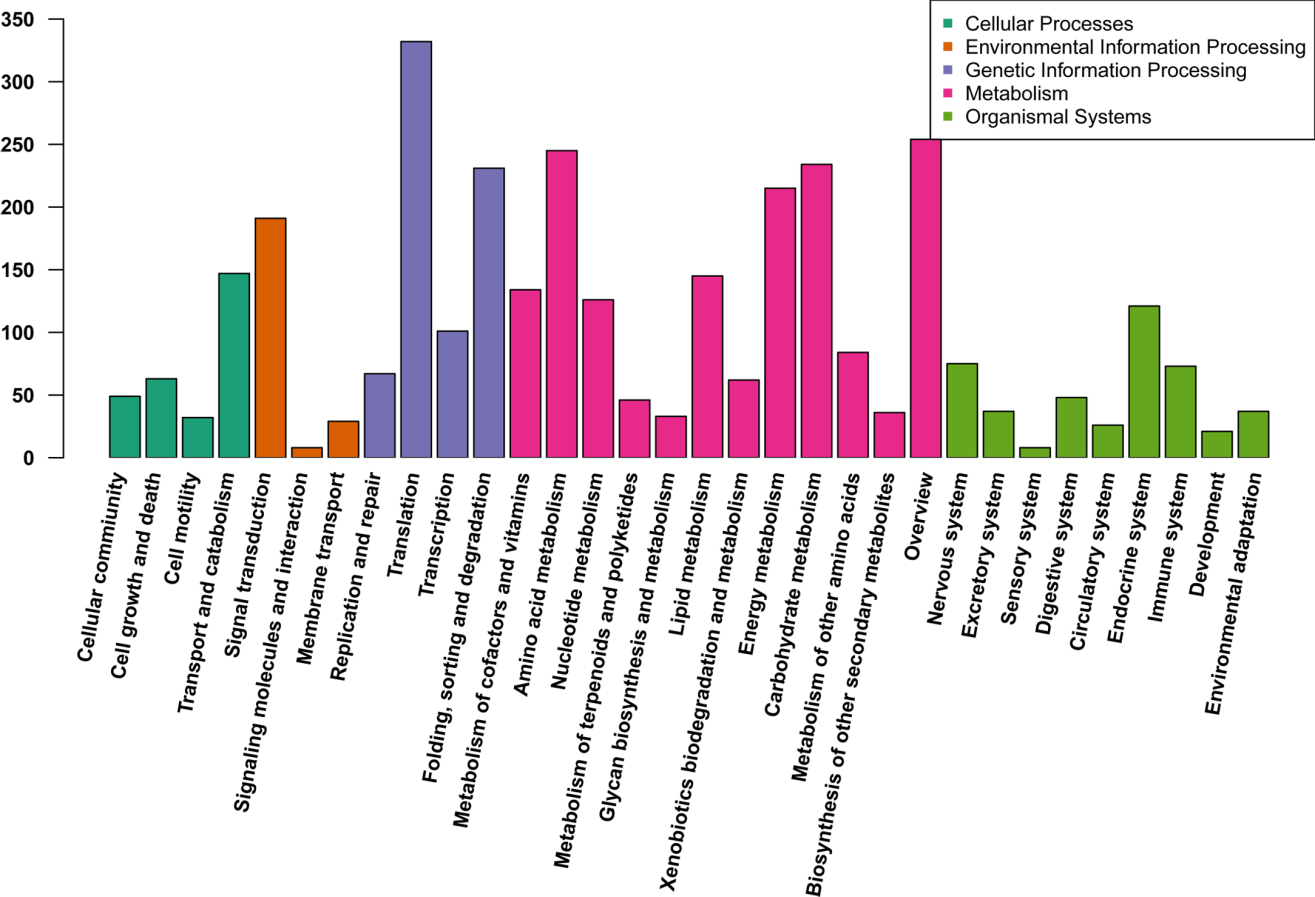
Functional classification of unigenes by using KEGG terms. The different colors represent the five KEGG groups: (A) cellular process; (B) environmental information processing; (C) genetic information processing; (D) metabolism; (E) organismal systems.

To further elucidate the physiological implications and interactions of the genes identified in our sequencing analysis, we blasted the unigenes against referenced canonical pathways in the KEGG database by using BLASTx with an E-value cutoff of 1e-5. We identified 4,587 (10.9%) unigenes that had at least one KEGG identifier in the 265 KEGG pathways. Among the metabolic pathways, genes related to amino acid, carbohydrate, and energy and lipid metabolism were highly represented in this set of *B*. *fuscopurpurea* unigenes (Fig 4). Most unigenes were involved in signaling pathways that play vital roles in responding and adapting to environmental stresses. For example, 41, 40, 33, 32, and 28 unigenes were predicted to encode the Ras-related C3 botulinum toxin substrate 1, GTPase KRas, MAPK1/3, MAP2K1, protein kinase A, and RAC serine/threonine-protein kinase AKT, respectively. Moreover, we found 273 unigenes (S7 Table) that participate in the fatty acid metabolic pathway, including fatty acid biosynthesis, fatty acid elongation, fatty acid degradation, arachidonic acid metabolism, linoleic acid metabolism, alpha-linolenic acid metabolism, and unsaturated fatty acid biosynthesis.

### Fatty acid content in *B*. *fuscopurpurea* changes in response to low temperature shock

**Fig 5:**
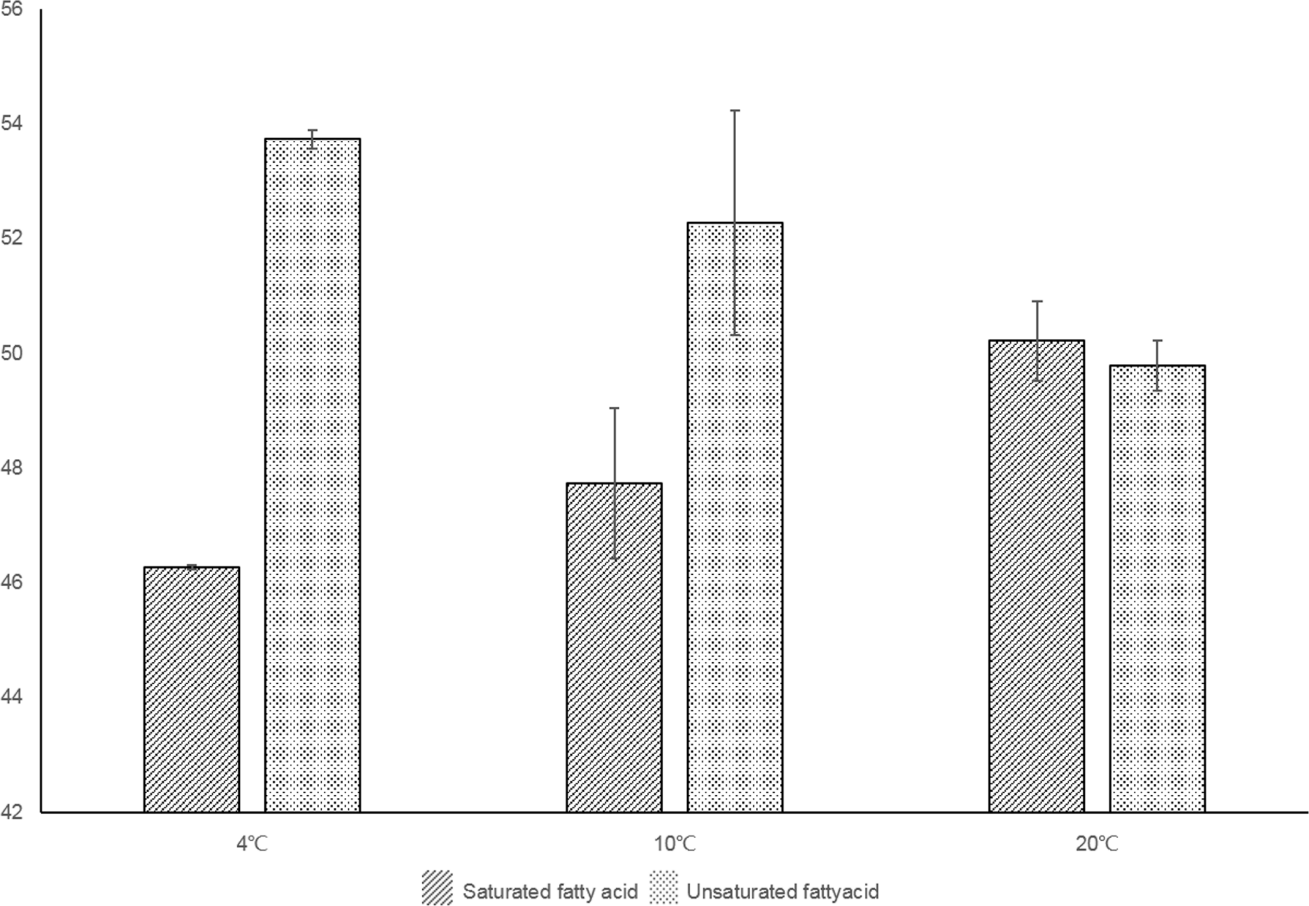
The saturated and unsaturated fatty acid content measured in *Bangia fuscopurpurea* grown under cold stress and normal conditions.

In plants, fatty acid modification in membrane lipids is one of the most important mechanisms that maintain membrane fluidity during extreme temperature changes. *B*. *fuscopurpurea* is an EPA-rich alga that can tolerate a broad range of environmental temperatures in its upper intertidal zone habitat. However, the metabolic dynamics of fatty acid composition and saturation under temperature-stressed conditions, especially the biological role of EPA metabolism, remain unknown. Therefore, in this study, fatty acid profiling as well as a gene expression analysis were simultaneously used to elucidate some of these metabolic processes. *B*. *fuscopurpurea* gametophytes were cultured under normal condition (20°C) and treated with cold temperature stress (4°C and 10°C) and collected for fatty acid analysis; about 23 major fatty acid species were thus identified. Among these, C16:0 was the most abundant fatty acid (44.9%), followed by C20:5 (25.0%) under normal temperature conditions. Although the total amount of fatty acids was stable in the temperature stress conditions, the extent of unsaturation of fatty acids increased significantly; in other words, the amount of unsaturated fatty acids was higher than that of saturated fatty acids under cold stress conditions (Fig 5). In particular, the C20:5 EPA content significantly increased (up to 30%) in gametophytes grown at 4°C; however, C16:0 decreased from 45.1% at 20°C to 41.0% at 4°C (Table 2). These results indicate that, when *B*. *fuscopurpurea* is exposed to low temperature, the PUFA amount increases rapidly in order to maintain cell membrane fluidity.

**Table 2:** The cellular fatty acid content in *Bangia fuscopurpurea* OUCPT-01 (%) grown under cold stress and normal conditions.

	4°C	10°C	20°C
C12:0	0.06±0.01	0.05±0.01	0.07±0.01
C14:0	1.32±0.02	1.31±0.12	1.28±0.07
C14:1	0.17±0.02	0.18±0.01	0.17±0.02
C16:0	40.98±0.11	42.59±1.14	45.05±0.68
C16:1ω7	0.54±0.05	0.27±0.03	0.30±0.02
C17:0	0.38±0.03	0.34±0.02	0.32±0.05
C17:1	0.15±0.02	0.15±0.02	0.17±0.03
C18:0	3.36±0.08	3.26±0.06	3.33±0.21
C18:1ω9	5.04±0.03	5.86±0.07	5.44±0.04
C18:1ω7	2.08±0.09	1.78±0.08	1.85±0.016
C18:2ω6	2.27±0.08	2.40±0.06	2.22±0.07
C18:3ω6	0.14±0.01	0.15±0.01	0.12±0.01
C18:3ω3	0.23±0.02	0.19±0.02	0.19±0.01
C18:4ω3	0.08±0.00	0.09±0.01	0.11±0.04
C20:1ω9	4.27±0.12	5.15±0.28	4.99±0.11
C20:2ω6	0.90±0.01	1.01±0.05	1.00±0.02
C20:3ω6	1.31±0.07	2.04±0.03	1.75±0.03
C20:3ω3	4.17±0.06	4.62±0.13	4.39±0.02
C20:4ω6	0.55±0.00	0.48±0.02	0.45±0.01
C20:4ω3	0.45±0.01	0.48±0.03	0.45±0.01
C20:5ω3	29.83±0.24	26.26±2.03	25.07±0.41
C22:1ω9	1.16±0.05	1.20±0.06	1.21±0.05
C22:6ω3	0.41±0.01	0.45±0.02	0.41±0.02
C20:3ω6	1.31±0.07	2.04±0.03	1.75±0.03
C20:3ω3	4.17±0.06	4.62±0.13	4.39±0.02
C20:4ω6	0.55±0.00	0.48±0.02	0.45±0.01
C20:4ω3	0.45±0.01	0.48±0.03	0.45±0.01
C20:5ω3	29.83±0.24	26.26±2.03	25.07±0.41
C22:1ω9	1.16±0.05	1.20±0.06	1.21±0.05
C22:6ω3	0.41±0.01	0.45±0.02	0.41±0.02

In plants and microalgae, EPA is synthesized from linoleic (18:2) and α-linolenic acid (18:3) via the ω-6 and ω-3 (as well as their bypass) pathways, respectively [26]. Both these pathways are involved in serial desaturation and elongation of fatty acids to form EPA (20:5). Further, delta5, delta6, and delta12 desaturases are the rate-limiting enzymes involved in highly unsaturated fatty acid (HUFA) biosynthesis [27–28]. These genes encoding the key nodes of the EPA biosynthesis pathway. The delta5 desaturase plays a role in EPA biosynthetic pathway and enables the cells to convert 20:3ω6 to 20:4ω6 [29]. To elucidate the mechanism of high EPA content in *B*. *fuscopurpurea*, we identified homologous genes encoding delta5 during EPA biosynthesis by conducting phylogenetic analysis (S2 Fig). Interestingly, up to five gene copies were present for delta5 desaturase (*comp53361*, *comp25166*, *comp5444*, *comp25177*, and *comp43330*) in the *B*. *fuscopurpurea* genome. Therefore, a phylogenetic analysis was conducted to infer the evolutionary origin of these genes. The *comp53361* and other red-algal homologs clustered with green algae, supporting their origin from a common primary endosymbiotic ancestor and independent evolution after the divergence of extant red and green algae. We excluded the possibility of an endosymbiotic gene transfer (EGT) from photosynthetic endosymbionts since delta5 desaturase was reported to be absent in cyanobacteria [30]. The most plausible explanation for this absence is the ancestral derivation from the eukaryotic host during primary endosymbiosis. In this clade, the red-algal delta5 desaturase genes showed strong affiliation with their counterparts from the heterokonta lineage. According to the hypothesis of secondary endosymbiosis suggesting that a eukaryotic cell engulfed a red algal plastid as well as the green algal plastid [31], we deduced that the red-algal delta5 desaturase gene might have been integrated into the nuclear genome via EGT and stably inherited thereafter. The other four delta5 desaturase genes in *B*. *fuscopurpurea* formed a sister group with choanoflagellates and animals. This further suggests that the repertoire of *B*. *fuscopurpurea* delta5 desaturase genes was derived from an ancestral heterotrophic flagellate that was the host for the primary endosymbiosis. Among delta5 desaturase genes, *comp25166* and *comp54444* were closely related to each other. The high similarity in their amino acid sequences (up to 80%) indicated the occurrence of gene duplication.

In the five delta5 desaturase homologs, we detected transcriptional variation for comp53361 under temperature stress. This gene exhibited significant elevation in transcriptional level under cold stress, especially at 4°C. Delta6 desaturase is responsible for converting 18:3ω3 (LNA) and 18:2ω6 (LA) to 18:4ω3 and 18:3ω6, respectively, and it is also involved in 22:6ω3 (DHA) synthesis from 20:5ω3 (EPA) [32]. The putative homolog of delta6 desaturase was also identified and showed a 4.36- and 1.56-fold elevation in transcriptional level at 4°C and 10°C, respectively, compared to that under normal temperature growth conditions. Delta6 elongase catalyzes the addition of the last two carbons onto C18 precursors to form C20 fatty chains. The transcriptional level of the delta6 elongase gene was up-regulated 2.0- and 2.61-fold at 4°C and 10°C, respectively. The simultaneous up-regulation of indicates their essential roles in the immediate channeling of C18:2 fatty chains into serial desaturation pathways to maintain membrane fluidity when *B*. *fuscopurpurea* encounters cold stress.

### Carbon flux distribution and EPA accumulation during temperature stress

**Fig 6:**
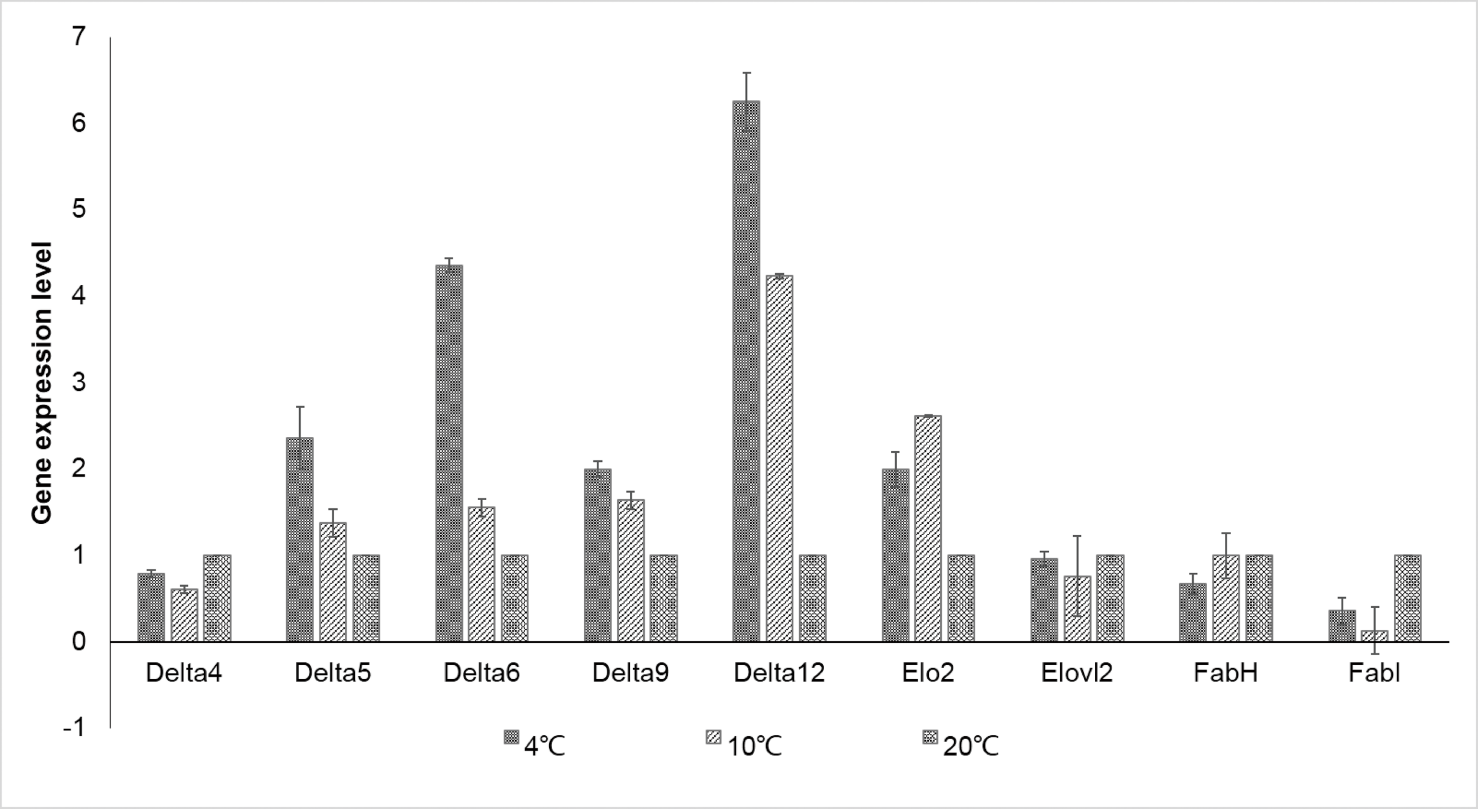
The relative transcriptional levels of eicosapntemacnioc acid (EPA) biosynthesis-related genes in *Bangia fuscopurpurea* grown under cold stress and normal conditions.

**Fig 7:**
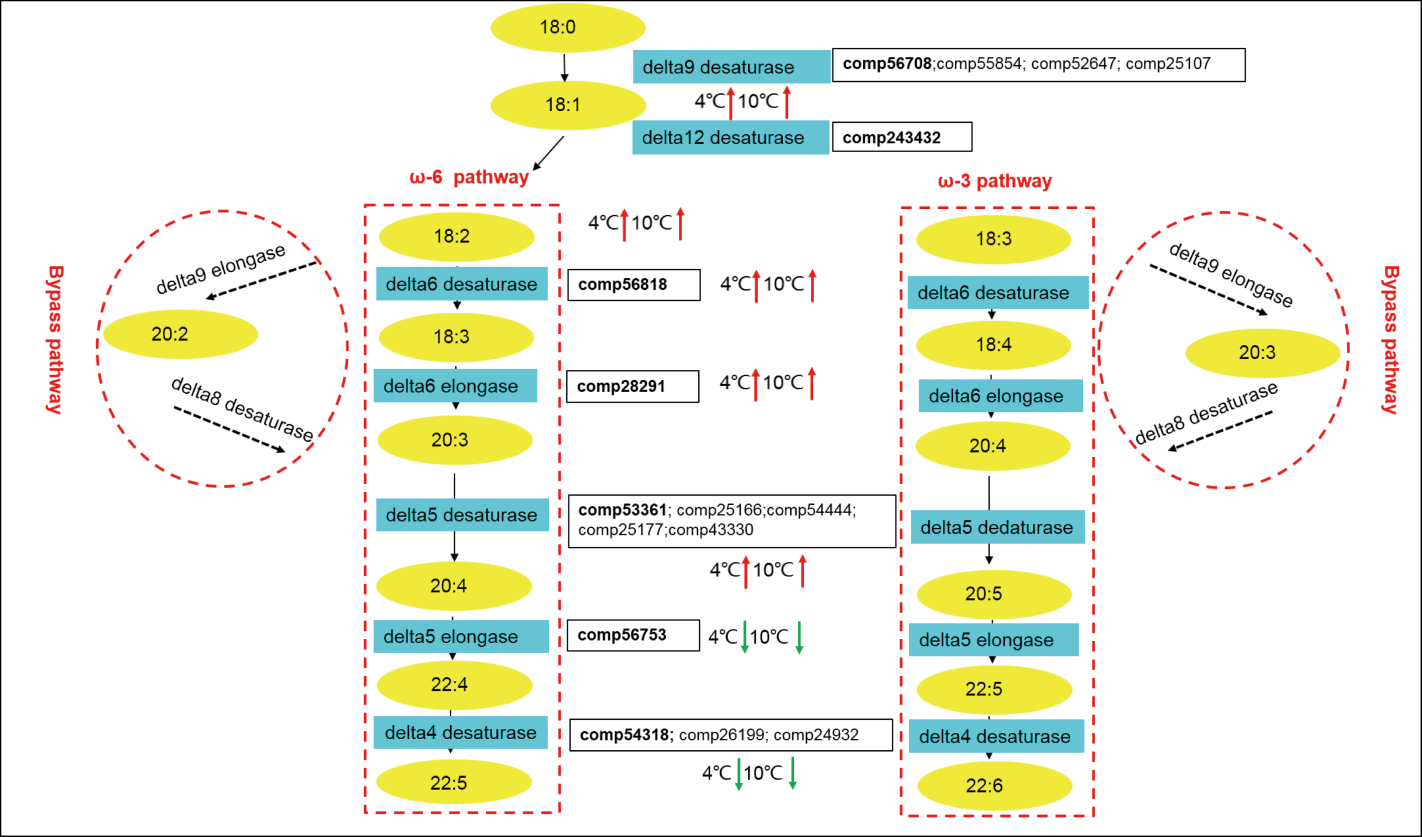
The eicosapntemacnioc acid (EPA) biosynthesis pathway in *Bangia fuscopurpurea*. The yellow ellipses represent fatty acid intermediates detected by fatty acid profiling. The blue rectangles represent enzymes that catalyze the reactions. Genes encoding the enzymes are listed in the neighboring blocks. Genes that were subjected to expression analysis are highlighted in red. The up-regulated and down-regulated transcriptional variations are marked by red and green arrows, respectively. Dashed arrows in the bypass pathways represent genes that were not identified in this study. ω-6 pathway is presented in the left dashed box, ω-3 pathway is present in the right dashed box and the bypass pathway is catalyzed by delta9 elongase and delta8 desaturase.

We identified the key genes involved in fatty acid biosynthesis pathways and investigated their transcriptional variation in *B*. *fuscopurpurea* grown under cold temperature stress. The C18:2 fatty acids are the carbon source for EPA biosynthesis. C18:2 could originate from either the desaturation of existing C18 fatty acid chains in membrane lipids or *de novo* synthesized fatty acids. Both 3-oxoacyl-[acyl-carrier-protein] synthase III (*FabH*) and enoyl-[acyl-carrier protein] reductase I (*FabI*) genes were down-regulated under low temperature conditions. The suppressed *de novo* fatty acid synthesis suggested that the existing C18:0 fatty acid chains from membrane lipids might be the major contributor to the carbon flux during PUFA accumulation. We also monitored the expression of genes encoding the two desaturases that sequentially catalyze the desaturation of C18:0. Both the genes encoding delta9 and delta12 desaturase were significantly elevated under cold stress conditions (Figs 6 and 7). However, the C18:2 content in *B*. *fuscopurpurea* grown under the temperature stress conditions were only slightly increased. One of the possible reasons could be that the newly produced C18:2 content was immediately channeled to polyunsaturated fatty acid biosynthesis (Table 2). Further, in the ω-6 and ω-3 pathways, EPA and its C20 precursors (e.g., C20:4) were transformed to DHA via sequential catalysis by delta5 elongase and delta4 desaturase. Under cold stress conditions, no obvious variation in the transcription of delta5 elongase was noted. However, the expression of the delta4 desaturase gene decreased slightly, suggesting a decline in DHA biosynthesis at cold temperatures. We did not detect any change in DHA content at 4°C; this might be due to the delayed response of metabolites compared to that of gene transcripts. Therefore, increased EPA content at cold temperatures might be attributed to the use of existing C18 fatty chains for serial desaturation facilitated by elevated transcription of delta9 and delta12 desaturase encoding genes and the decline in the consumption of newly produced EPA.

## Discussion

### The genetic mechanism of EPA production in *B*. *fuscopurpurea*

Polyunsaturated fatty acids, especially EPA (20:5n3), an ω-3 polyunsaturated fatty acid with a broad range of potentially beneficial effects for humans, are essential fatty acids and must be obtained from dietary sources [2]. At present, EPA is mainly obtained from fish oil [33], although it also has been verified that microalgae and macroalgae contain high levels of PUFAs similar to that in fish oil [34]. Previous studies have characterized several synthetic pathways for EPA in algae. In the diatom *P*. *tricornutum*, pulse-chase experiments with various C14 molecules showed that four pathways contribute to EPA production; in contrast, in *Spirulina platensis*, only the ω-6 pathway was identified [35–36]. However, the bypass pathway for C20 synthesis that is catalyzed by delta9 elongase and delta8 desaturase was detected in *Isochrysis galbana* [37]. In our study, the genes and metabolites in ω-6 and ω-3 pathways were all detected in *B*. *fuscopurpurea*. Although the genes in the bypass pathways remain unknown (e.g., delta9 elongase and delta8 desaturase), intermediate metabolites, including 20:3ω3 and 20:2ω6, in all of the three pathways were detected, suggesting that they all contribute to EPA production as they do in *P*. *tricornutum*. Therefore, three biosynthetic pathways are presumed to exist for EPA in *B*. *fuscopurpurea* that include the classical ω-6, ω-3, and bypass pathways, which might contribute to the increase in EPA biosynthesis.

The EPA biosynthesis pathways include a serial process of desaturation and elongation of precursor fatty acids such as C18:2 and C18:3. Delta5 desaturase is reported to be one of the key enzymes that control the production of EPA [29]. In *B*. *fuscopurpurea*, five putative paralogs of delta5 desaturase are present. Even in *Nannochloropsis oceanica*, a microalga with high lipid and EPA content, only three gene copies for delta5 desaturase are present [38]. Further, *Dictyostelium discoideum* has only two delta5 desaturase genes [39]. We speculate that the diverse biosynthetic pathways and expanded gene repertoire for fatty chain desaturation provide *B*. *fuscopurpurea* with high EPA production abilities. Moreover, one of the delta5 desaturase genes was significantly upregulated concomitantly with the accumulation of EPA under cold stress. Subsequently, the up-regulation of one delta5 desaturase paralogous gene was consistent with a 4.76% increase in EPA content. This gene could be a potential target for subsequent gene engineering to maximize EPA production. The transcriptional patterns of the other four gene copies need to be investigated in the future.

### The molecular mechanism of fatty acid modification in response to cold stress

Previous studies have indicated that the higher the content of unsaturated fatty acids in cell membranes, the easier it is for organisms to resist damage caused by cold stress [40]. Thus, the content of unsaturated fatty acids could be used as an indicator for investigating tolerance to cold stress [41].

Abiotic stress-induced changes in the fatty acid composition of plant membrane lipids mainly occur through the regulated activities of fatty acid desaturases at both transcriptional and post-transcriptional levels [42]. As in other red algae [43], we found that the content of polyunsaturated fatty acids increased, whereas that of saturated fatty acids reduced under cold stress in *B*. *fuscopurpurea*. We detected transcriptional variation of genes encoding three key enzymes (delta5 and delta6 desaturase, delta5 elongase) that catalyze the transformation of C18:2 and C18:3 fatty acids into EPA. All of these genes exhibited simultaneous up-regulation at the transcriptional level, suggesting the existence of an elaborate regulatory mechanism that could be induced by cold stress. The gene expression patterns presented in this study are similar to those reported in *Synechocystis* sp. PCC 6803, *Chlorella minutissima*, and *Nitzschia closterium*; the levels of mRNAs that encode unpolyunsaturated fatty acids increased when the conditions changed from high to low temperature [13,44]. Further analysis of the promoter and *cis*-regulatory elements for these genes might enable better definition of the molecular mechanism of temperature-sensitive fatty acid biosynthesis.

## Supporting information

S1 Fig The macroscopic and microscopic photos of *Bangia fuscopurpurea* gametophytes. (TIF)

S2 Fig Phylogenetic relationships between the predicted desaturases of *B. fuscopurpurea* and that from other desaturase genes. (TIF)

S1 Table Various stress-treated *B. fuscopurpurea* samples used for transcriptome sequencing. (DOC)

S2 Table List of organisms and desaturase protein sequences. (DOC)

S3 Table RIN quality values for RNAs used for qPCR gene expression. (DOC)

S4 Table Primers of the 12 *B. fuscopurpurea* genes used for quantitative real-time PCR analysis. (DOC)

S5 Table Raw data for qPCR. (DOC)

S6 Table Annotation results of *B. fuscopurpurea* genes/proteins based on different databases. (DOC)

S7 Table The 273 unigenes related to fatty acid biosynthesis pathways. (DOC)
