# Supplementary figures and images for: Integrating transcriptomics and metabolomics to characterize the regulation of EPA biosynthesis in response to cold stress in seaweed Bangia fuscopurpurea
Source: PLoS One. 2017 Dec 14;12(12):e0186986. doi: 10.1371/journal.pone.0186986 (PMC5730106; doi:10.1371/journal.pone.0186986)

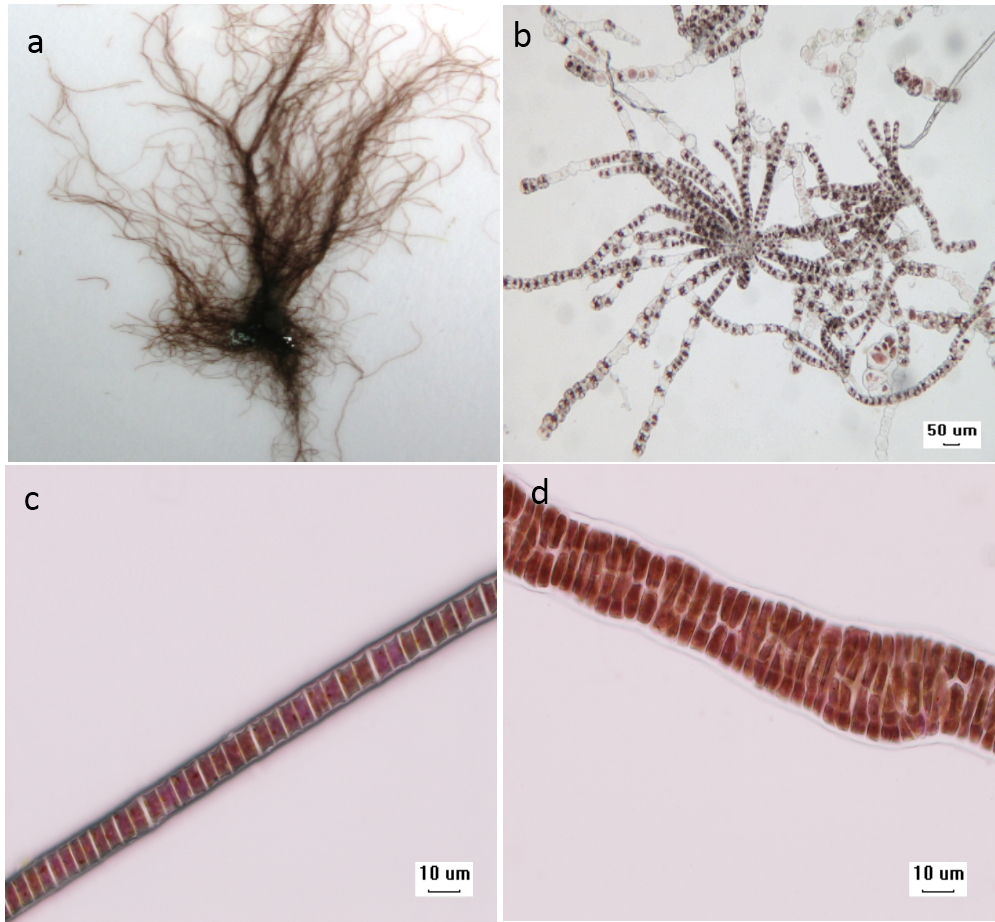

Supplement: S1 Fig — (TIF) [file pone.0186986.s001.tif]

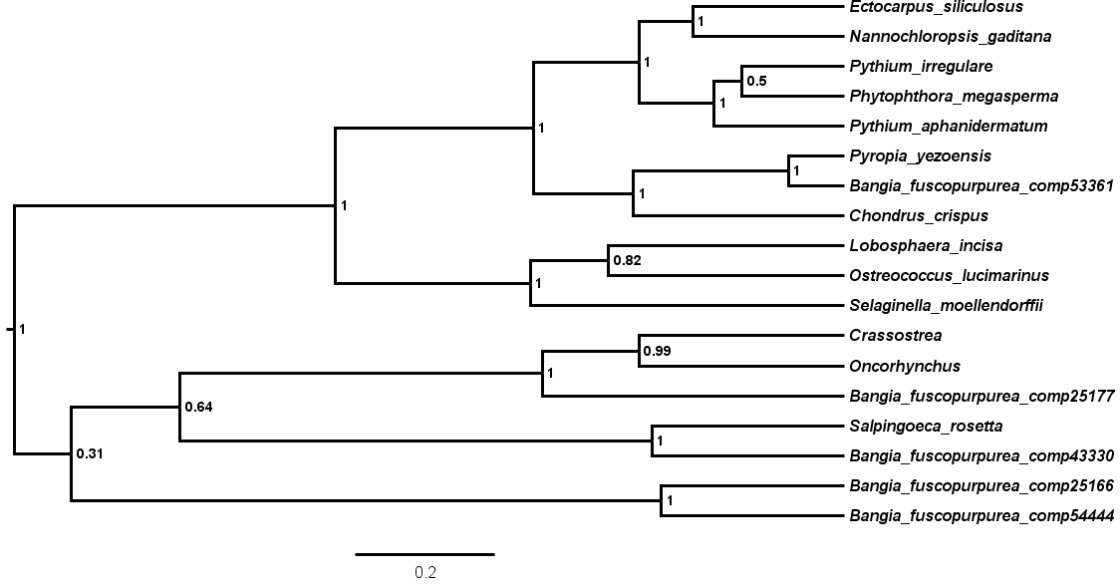

Supplement: S2 Fig — (TIF) [file pone.0186986.s002.tif]
