# Supplementary material for: Integrating transcriptomics and metabolomics to characterize the regulation of EPA biosynthesis in response to cold stress in seaweed Bangia fuscopurpurea
Source: PLoS One. 2017 Dec 14;12(12):e0186986. doi: 10.1371/journal.pone.0186986 (PMC5730106; doi:10.1371/journal.pone.0186986)
Supplement: S1 Table — (DOC) [file pone.0186986.s003.doc]

Table S1 Different cultivated conditions of *B. fuscopurpurea* for cDNA library construction

| Samples |  | Cultivation conditions |
| --- | --- | --- |
| Gametophyte |  | 20°C, 50～60μmol photons m^-2^s^-1^, 12L:12D, Salinity: 33‰ |
| Gametophyte |  | 4°C, 50～60μmol photons m^-2^s^-1^, 12L:12D,4H, Salinity: 33‰ |
| Gametophyte |  | 10°C, 50～60μmol photons m^-2^s^-1^, 12L:12D,4H, Salinity: 33‰ |
| Gametophyte |  | 30°C, 50～60μmol photons m^-2^s^-1^, 12L:12D,4H, Salinity: 33‰ |
| Gametophyte |  | 20°C, 400～500μmol photons m^-2^s^-1^, 12L:12D,4H, Salinity: 33‰ |
| Gametophyte |  | 20°C, 0μmol photons m^-2^s^-1^, 12L:12D,4H, Salinity: 33‰ |
| Gametophyte |  | Materials were collected before 1 hour of the light cylce, Salinity: 33‰ |
| Gametophyte |  | Materials were collected before 1 hour of the dark cylce, Salinity: 33‰ |
| Gametophyte |  | Materials were collected after 3 hours of the light cylce, Salinity: 33‰ |
| Gametophyte |  | 20°C, 50～60μmol photons m^-2^s^-1^, 12L:12D, Salinity: 66‰ |
| Gametophyte |  | 20°C, 50～60μmol photons m^-2^s^-1^, 12L:12D, Salinity: 17‰ |
| Gametophyte |  | 20°C, 50～60μmol photons m^-2^s^-1^, 12L:12D, Salinity: 0‰ |
| Gametophyte |  | Drought conditions in controlled room, 20°C, water content at 30% |
| Gametophyte |  | Drought conditions in controlled room, 20°C, water content at 70% |
| Gametophyte |  | Rehydration from the materials with water content 30% for 40min |
| Gametophyte  Gametophyte |  | Rehydration from the materials with water content 70% for 40min  20°C, 50～60μmol photons m-2s-1, 12L:12D, Salinity: 33‰, lack of nutrition cultured for 5 days |
| Gametophyte |  | Drought conditions in desiccant for 1h, 20°C, water content at 30% |
| Gametophyte |  | Drought conditions in desiccant for 2.5h, 20°C, water content at 70% |
