# Supplementary material for: Integrating transcriptomics and metabolomics to characterize the regulation of EPA biosynthesis in response to cold stress in seaweed Bangia fuscopurpurea
Source: PLoS One. 2017 Dec 14;12(12):e0186986. doi: 10.1371/journal.pone.0186986 (PMC5730106; doi:10.1371/journal.pone.0186986)
Supplement: S2 Table — (DOC) [file pone.0186986.s004.doc]

Table S2 List of organisms and desaturase protein sequences

| Organism | Accession NO. | Desaturase name |
| --- | --- | --- |
| *Bangia fuscopurpurea* comp25166 | In this study | Desaturase 5 |
| *Bangia fuscopurpurea* comp54444 | In this study | Desaturase 5 |
| *Bangia fuscopurpurea* comp25177 | In this study | Desaturase 5 |
| *Bangia fuscopurpurea* comp43330 | In this study | Desaturase 5 |
| *Bangia fuscopurpurea* comp53361 | In this study | Desaturase 5 |
| *Pyropia yezoensis* | ACB11556.1 | Desaturase 5 |
| *Chondrus crispus* | XP 005713430.1 | Desaturase 5 |
| *Pythium irregulare* | AAL13311.1 | Desaturase 5 |
| *Nannochloropsis gaditana* | EWM23841.1 | Desaturase 5 |
| *Pythium aphanidermatum* | AGS55977.1 | Desaturase 5 |
| *Phytophthora megasperma* | CAD53323.1 | Desaturase 5 |
| *Ectocarpus siliculosus* | CBJ29129.1 | Desaturase 5 |
| *Lobosphaera incisa* | ADB81956.1 | Desaturase 5 |
| *Ostreococcus lucimarinus* | XP_001420855.1 | Desaturase 5 |
| *Salpingoeca rosetta* | XP_004998044 | Desaturase 5 |
| *Selaginella moellendorffii* | XP_002988231.1 | Desaturase 5 |
