# Supplementary material for: Integrating transcriptomics and metabolomics to characterize the regulation of EPA biosynthesis in response to cold stress in seaweed Bangia fuscopurpurea
Source: PLoS One. 2017 Dec 14;12(12):e0186986. doi: 10.1371/journal.pone.0186986 (PMC5730106; doi:10.1371/journal.pone.0186986)
Supplement: S3 Table — (DOC) [file pone.0186986.s005.doc]

Table S4 RIN quality values for RNAs used for qPCR gene expression

| Sample ID | Conc. (µg/µL) | O.D. 260/280 | O.D. 260/230 | Amount (µg) | rRNA 28S/18S | RIN |
| --- | --- | --- | --- | --- | --- | --- |
| 20°C-1 | 0.11 | 1.95 | 1.50 | 2.75 | 1.1 | 7.0 |
| 20°C-2 | 0.06 | 1.90 | 1.76 | 2.89 | 0.8 | 7.1 |
| 20°C-3 | 0.09 | 1.97 | 1.58 | 1.11 | 1.1 | 7.3 |
| 4°C-1 | 0.05 | 2.19 | 1.52 | 1.26 | 0.7 | 7.0 |
| 4°C-2 | 0.11 | 2.16 | 1.56 | 1.60 | 1.3 | 7.5 |
| 4°C-3 | 0.07 | 2.15 | 1.57 | 1.48 | 1.0 | 7.6 |
| 10°C-1 | 0.03 | 2.18 | 1.59 | 1.67 | 1.0 | 7.7 |
| 10°C -2 | 0.09 | 2.21 | 1.56 | 1.80 | 1.1 | 7.9 |
| 10°C -3 | 0.07 | 2.20 | 1.69 | 3.09 | 1.0 | 7.4 |
