# Supplementary material for: Integrating transcriptomics and metabolomics to characterize the regulation of EPA biosynthesis in response to cold stress in seaweed Bangia fuscopurpurea
Source: PLoS One. 2017 Dec 14;12(12):e0186986. doi: 10.1371/journal.pone.0186986 (PMC5730106; doi:10.1371/journal.pone.0186986)
Supplement: S4 Table — (DOC) [file pone.0186986.s006.doc]

Table S3 Primers of genes used for quantitative real-time PCR in *B. fuscopurpurea*

|  | **Forward primer (**5’-3’**)** | **Reversed primer (**5’-3’**)** | **Efficiency** |
| --- | --- | --- | --- |
| Tublin | GTGGACTGGGTGATGGGG | ACAGCGTGGCGGACAAAA | 103% |
| GAPDH | CTGGTGAGGCACTTTGGAA | AAGGAGGAGGACTGATGGG | 105% |
| Delta12 | CACCCAGAGGCAAACCC | ATGGACGGGCAGTAGGG | 96% |
| Delta9 | GGAGAAGTTGGAATGAGGC | AGTAGGCGGAACATAAGAG | 91% |
| Delta4 | CGGGTTGTCCGAAAAGGC | AGGTGAGTATGCGGGTGG | 92% |
| Delta6 | GTTCCCCACCGTCTCGCAGTA | CCGTCTTCCGTCCCACCACA | 104% |
| Delta5 | AGTTATTGATTGGGGTGA | ATTCTTTGCGTGTTTCTTG | 99% |
| FabH | AATACATCTGCCGCTTCG | TCTCGTCATCCGCACTAA | 94% |
| FabI | GAGGGACCAGGAGAAGC | CACGGAGGATACAAAACC | 100% |
| ELO2 | GTGGTCGCAGATTGTTGA | ACGGCATAGGCGTAGGAG | 97% |
| Elvolv2 | GACTTTCCCGATGTCCTCA | CCTTGCCCTTGTTCTTCAC | 98% |
