# Supplementary material for: Integrating transcriptomics and metabolomics to characterize the regulation of EPA biosynthesis in response to cold stress in seaweed Bangia fuscopurpurea
Source: PLoS One. 2017 Dec 14;12(12):e0186986. doi: 10.1371/journal.pone.0186986 (PMC5730106; doi:10.1371/journal.pone.0186986)
Supplement: S5 Table — (DOC) [file pone.0186986.s007.doc]

Table SX Raw data on cycle thresholds and their statistical analysis

1. Cycle thresholds for the amplification efficiency calculation

| Gene name | Cycle thresholds (replicates 1-3) | | | Average |
| --- | --- | --- | --- | --- |
| Tublin | 9.91 | 10.29 | 9.8 | 10.00 |
|  | 13.58 | 13.7 | 13.67 | 13.65 |
|  | 16.75 | 17.31 | 16.78 | 16.95 |
|  | 21.36 | 21.58 | 21.48 | 21.47 |
|  | 22.85 | 22.85 | 22.92 | 22.87 |
|  | 25.99 | 26.58 | 26.59 | 26.39 |
|  |  |  |  |  |
| GAPDH | 14.51 | 15.27 | 15.54 | 15.11 |
|  | 19.27 | 19.22 | 19.46 | 19.32 |
|  | 22.33 | 22.4 | 22.25 | 22.33 |
|  | 25.69 | 25.68 | 25.56 | 25.64 |
|  | 27.71 | 28.34 | 27.95 | 28.00 |
|  |  |  |  |  |
| Delta12 | 7.8 | 7.69 | 7.76 | 7.75 |
|  | 11.27 | 11.29 | 11.56 | 11.37 |
|  | 14.83 | 14.88 | 14.99 | 14.90 |
|  | 18.48 | 18.51 | 18.57 | 18.52 |
|  | 21.41 | 21.95 | 21.23 | 21.53 |
|  | 24.73 | 24.8 | 24.86 | 24.80 |
|  |  |  |  |  |
| Delta9 | 7.52 | 7.27 | 7.365 | 7.39 |
|  | 10.7 | 10.6 | 10.11 | 10.47 |
|  | 14.54 | 14.52 | 14.32 | 14.46 |
|  | 17.72 | 17.62 | 17.27 | 17.54 |
|  | 21.81 | 21.84 | 21.25 | 21.63 |
|  |  |  |  |  |
| Delta4 | 17.67 | 17.83 | 17.25 |  |
|  | 21.36 | 21.46 | 21.14 | 21.32 |
|  | 24.2 | 23.86 | 23.83 | 23.96 |
|  | 28.83 | 29.44 | 29.25 | 29.17 |
|  | 31.27 | 31.33 | 31.17 | 31.26 |
|  | 17.67 | 17.83 | 17.25 | 17.58 |
| Delta6 |  |  |  |  |
|  | 6.94 | 6.86 | 6.89 | 6.90 |
|  | 9.79 | 9.93 | 9.89 | 9.87 |
|  | 12.91 | 12.89 | 12.93 | 12.91 |
|  | 16.65 | 16.75 | 16.71 | 16.70 |
|  | 19.63 | 19.75 | 19.67 | 19.68 |
| Delta5 |  |  |  |  |
|  | 8.34 | 7.9 | 8.1 | 8.11 |
|  | 10.83 | 10.85 | 10.84 | 10.84 |
|  | 13.61 | 13.6 | 13.61 | 13.61 |
|  | 16.85 | 17.17 | 17.13 | 17.05 |
|  | 20.71 | 20.8 | 20.73 | 20.75 |
|  | 24.53 | 25.06 | 24.91 | 24.83 |
|  |  |  |  |  |
| FabH |  |  |  |  |
|  | 12.48 | 12.95 | 12.72 | 12.72 |
|  | 15.33 | 15.35 | 15.34 | 15.34 |
|  | 19.76 | 19.79 | 19.78 | 19.78 |
|  | 21.61 | 21.73 | 21.67 | 21.67 |
|  | 26.64 | 26.67 | 26.66 | 26.66 |
| FabI |  |  |  |  |
|  | 7.74 | 7.73 | 7.72 | 7.73 |
|  | 10.87 | 10.8 | 10.79 | 10.82 |
|  | 14.82 | 14.98 | 14.91 | 14.90 |
|  | 19.23 | 19.22 | 19.24 | 19.23 |
|  | 21.53 | 21.36 | 21.45 | 21.45 |
|  | 23.74 | 23.71 | 23.73 | 23.73 |
|  |  |  |  |  |
| ELO2 |  |  |  |  |
|  | 10.5 | 9.55 | 10.02 | 10.02 |
|  | 12.65 | 13.4 | 13.12 | 13.06 |
|  | 16.61 | 16.56 | 16.57 | 16.58 |
|  | 20.36 | 20.34 | 20.33 | 20.34 |
|  | 23.97 | 24.2 | 24.12 | 24.10 |
|  | 26.44 | 26.56 | 26.45 | 26.48 |
|  |  |  |  |  |
| Elvolv2 |  |  |  |  |
|  | 7.84 | 7.46 | 7.66 | 7.65 |
|  | 10.52 | 10.55 | 10.51 | 10.53 |
|  | 13.99 | 13.92 | 14.02 | 13.98 |
|  | 16.96 | 16.88 | 16.82 | 16.89 |
|  | 20.37 | 20.3 | 20.34 | 20.34 |
|  | 24.63 | 24.96 | 24.8 | 24.80 |

1. Cycle thresholds of the raw data for gene expression

| 4℃ | Replicate 1 | Replicate 2 | Replicate 3 | Average |
| --- | --- | --- | --- | --- |
| Delta4 | 27.40 | 27.38 | 27.39 | 27.39 |
| Delta5 | 29.03 | 28.98 | 28.92 | 28.98 |
| Delta6 | 26.38 | 26.37 | 26.39 | 26.38 |
| Delta9 | 24.30 | 24.22 | 24.21 | 24.24 |
| Delta12 | 30.11 | 30.09 | 30.11 | 30.10 |
| Elo2 | 25.77 | 25.87 | 25.84 | 25.83 |
| Elovl2 | 29.98 | 30.07 | 30.05 | 30.03 |
| FabH | 27.91 | 27.92 | 27.96 | 27.93 |
| FabI | 30.02 | 30.02 | 30.07 | 30.04 |
|  |  |  |  |  |
| 10℃ | Replicate 1 | Replicate 2 | Replicate 3 | Average |
| Delta4 | 27.01 | 27.03 | 27.01 | 27.02 |
| Delta5 | 28.17 | 28.15 | 28.28 | 28.20 |
| Delta6 | 24.91 | 24.89 | 24.88 | 24.89 |
| Delta9 | 23.97 | 23.95 | 23.92 | 23.95 |
| Delta12 | 29.55 | 29.52 | 29.54 | 29.54 |
| Elo2 | 26.20 | 26.21 | 26.24 | 26.22 |
| Elovl2 | 29.65 | 29.72 | 29.72 | 29.70 |
| FabH | 28.48 | 28.56 | 28.46 | 28.51 |
| FabI | 28.57 | 28.62 | 28.56 | 28.58 |
|  |  |  |  |  |
| 20℃ | Replicate 1 | Replicate 2 | Replicate 3 | Average |
| Delta4 | 26.78 | 26.80 | 26.99 | 26.86 |
| Delta5 | 29.50 | 30.14 | 32.24 | 30.63 |
| Delta6 | 26.85 | 27.41 | 26.74 | 27.00 |
| Delta9 | 18.55 | 18.95 | 18.37 | 18.62 |
| Delta12 | 30.84 | 30.95 | 30.25 | 30.68 |
| Elo2 | 28.69 | 28.58 | 28.81 | 28.69 |
| Elovl2 | 32.99 | 31.71 | 38.39 | 34.36 |
| FabH | 31.19 | 29.66 | 30.77 | 30.54 |
| FabI | 26.44 | 26.59 | 26.54 | 26.52 |

1. Calculation for gene expression

|  | Delta4 | Delta5 | Delta6 | Delta9 | Delta12 | Elo2 | Elovl2 | FabH | FabI |
| --- | --- | --- | --- | --- | --- | --- | --- | --- | --- |
| 4℃ | 27.39 | 28.98 | 26.38 | 24.24 | 30.10 | 25.83 | 30.03 | 27.93 | 30.04 |
| 10℃ | 27.02 | 28.20 | 24.89 | 23.95 | 29.54 | 26.22 | 29.70 | 28.51 | 28.58 |
| 20℃ | 26.86 | 30.63 | 27.00 | 18.62 | 30.68 | 28.69 | 34.36 | 30.54 | 26.52 |
|  |  |  |  |  |  |  |  |  |  |
|  | △Ct |  |  |  |  |  |  |  |  |
|  | Delta4 | Delta5 | Delta6 | Delta9 | Delta12 | Elo2 | Elovl2 | FabH | FabI |
| 4℃ | 0.54 | -1.65 | -0.62 | 5.62 | -0.58 | -2.86 | -4.33 | -2.61 | 3.52 |
| 10℃ | 0.16 | -2.43 | -2.11 | 5.33 | -1.14 | -2.47 | -4.67 | -2.03 | 2.06 |
| 20℃ | 0.88 | -2.89 | -2.74 | 4.62 | -3.22 | -3.86 | -4.27 | -2.03 | 5.01 |
|  |  |  |  |  |  |  |  |  |  |
|  | △△Ct |  |  |  |  |  |  |  |  |
|  | Delta4 | Delta5 | Delta6 | Delta9 | Delta12 | Elo2 | Elovl2 | FabH | FabI |
| 4℃ | -0.35 | 1.24 | 2.12 | 1.00 | 2.64 | 1.00 | -0.06 | -0.58 | -1.49 |
| 10℃ | -0.72 | 0.46 | 0.64 | 0.71 | 2.08 | 1.38 | -0.40 | 0.00 | -2.95 |
| 20℃ | 0.00 | 0.00 | 0.00 | 0.00 | 0.00 | 0.00 | 0.00 | 0.00 | 0.00 |
|  |  |  |  |  |  |  |  |  |  |
|  | 2^-△△Ct^ |  |  |  |  |  |  |  |  |
|  | Delta4 | Delta5 | Delta6 | Delta9 | Delta12 | Elo2 | Elovl2 | FabH | FabI |
| 4℃ | 0.79 | 2.36 | 4.36 | 2.00 | 6.25 | 2.00 | 0.96 | 0.67 | 0.36 |
| 10℃ | 0.61 | 1.38 | 1.56 | 1.64 | 4.23 | 2.61 | 0.76 | 1.00 | 0.13 |
| 20℃ | 1.00 | 1.00 | 1.00 | 1.00 | 1.00 | 1.00 | 1.00 | 1.00 | 1.00 |
