# Supplementary material for: Integrating transcriptomics and metabolomics to characterize the regulation of EPA biosynthesis in response to cold stress in seaweed Bangia fuscopurpurea
Source: PLoS One. 2017 Dec 14;12(12):e0186986. doi: 10.1371/journal.pone.0186986 (PMC5730106; doi:10.1371/journal.pone.0186986)
Supplement: S6 Table — (DOC) [file pone.0186986.s008.doc]

Table S4 Annotation results of the *B. fuscopurpurea* based on different database

| Anno Database | Annotated_Number | length>=300bp&&<1000bp | length>=1000bp |
| --- | --- | --- | --- |
| nr_Annotation | 8134 | 4005 | 1558 |
| Kegg_Annotation | 6829 | 3345 | 1285 |
| Swissport_Annotation | 5688 | 2754 | 1078 |
| KOG_Annotation | 5552 | 2688 | 1045 |
| ipr_Annotation | 8708 | 4450 | 2164 |
| GO_Annotation | 3164 | 1548 | 682 |
| TrEMBL_Annotation | 7227 | 3532 | 1335 |
| All_Annotation | 11286 | 5637 | 2336 |
