# Supplementary material for: Integrating transcriptomics and metabolomics to characterize the regulation of EPA biosynthesis in response to cold stress in seaweed Bangia fuscopurpurea
Source: PLoS One. 2017 Dec 14;12(12):e0186986. doi: 10.1371/journal.pone.0186986 (PMC5730106; doi:10.1371/journal.pone.0186986)
Supplement: S7 Table — (DOC) [file pone.0186986.s009.doc]

Table SX 273 unigenes related to fatty acid biosynthesis pathways

| Gene_ID | Gene_annotation |
| --- | --- |
| comp10764_c0_seq1 | Short-chain acyl-CoA dehydrogenase |
| comp111239_c0_seq1 | Triglyceride lipase-cholesterol esterase |
| comp113173_c0_seq1 | Short-chain acyl-CoA dehydrogenase |
| comp113389_c0_seq1 | CYP710A; cytochrome P450, family 710, subfamily A |
| comp114206_c0_seq1 | Short-chain acyl-CoA dehydrogenase |
| comp122868_c0_seq1 | Predicted phospholipase |
| comp123543_c0_seq1 | Phosphomannomutase |
| comp124157_c0_seq1 | Triglyceride lipase-cholesterol esterase |
| comp125692_c0_seq1 | scoA; 3-oxoacid CoA-transferase subunit A |
| comp128817_c0_seq1 | Mitochondrial/plastidial beta-ketoacyl-ACP reductase |
| comp131754_c0_seq1 | Cis-prenyltransferase |
| comp138151_c0_seq1 | Phosphatidylserine decarboxylase |
| comp143971_c0_seq1 | Oxidosqualene-lanosterol cyclase and related proteins |
| comp146669_c0_seq1 | Very-long-chain acyl-CoA dehydrogenase |
| comp146702_c0_seq1 | Acyl-CoA oxidase |
| comp158228_c0_seq1 | Acyl-CoA-binding protein |
| comp160977_c0_seq1 | Carnitine O-acyltransferase CRAT |
| comp164124_c0_seq1 | Long-chain acyl-CoA synthetases (AMP-forming) |
| comp18170_c0_seq1 | Acyl-CoA oxidase |
| comp183428_c0_seq1 | Isoamyl acetate-hydrolyzing esterase |
| comp185588_c0_seq1 | Sphingosine kinase, involved in sphingolipid metabolism |
| comp188383_c0_seq1 | Oxidosqualene-lanosterol cyclase and related proteins |
| comp189272_c0_seq1 | Enoyl-CoA hydratase/isomerase |
| comp192477_c0_seq1 | 3-oxoacyl CoA thiolase |
| comp206649_c0_seq1 | Diacylglycerol kinase |
| comp210170_c0_seq1 | Steroid reductase required for elongation of the very long chain fatty acids |
| comp210913_c0_seq1 | Sterol reductase/lamin B receptor |
| comp211141_c0_seq1 | Peroxisomal long-chain acyl-CoA transporter, ABC superfamily |
| comp216060_c0_seq1 | Very long-chain acyl-CoA synthetase/fatty acid transporter |
| comp23729_c0_seq1 | Hydroxyacyl-CoA dehydrogenase/enoyl-CoA hydratase |
| comp240092_c0_seq1 | Enoyl-CoA isomerase |
| comp243432_c0_seq1 | FAD2; omega-6 fatty acid desaturase (delta-12 desaturase) |
| comp247078_c0_seq1 | Triglyceride lipase-cholesterol esterase |
| comp247688_c0_seq1 | Lysophosphatidic acid acyltransferase LPAAT and related acyltransferases |
| comp24932_c0_seq1 | Fatty acid desaturase |
| comp25047_c0_seq1 | Short-chain acyl-CoA dehydrogenase |
| comp25107_c0_seq1 | Fatty acid desaturase |
| comp25137_c0_seq1 | Enoyl-CoA hydratase |
| comp25166_c0_seq1 | delta5 fatty acid desaturase |
| comp25177_c0_seq1 | Delta 6-fatty acid desaturase/delta-8 sphingolipid desaturase |
| comp25756_c0_seq1 | scoA; 3-oxoacid CoA-transferase subunit A |
| comp26199_c0_seq1 | Fatty acid desaturase |
| comp26250_c0_seq1 | Very long-chain acyl-CoA synthetase/fatty acid transporter |
| comp26369_c0_seq1 | gpsA; glycerol-3-phosphate dehydrogenase (NAD(P)+) |
| comp26634_c0_seq1 | Acyl-CoA oxidase |
| comp27154_c0_seq1 | KCS; 3-ketoacyl-CoA synthase [EC:2.3.1.199] |
| comp27310_c0_seq1 | PHS1, PAS2; very-long-chain (3R)-3-hydroxyacyl-[acyl-carrier protein] dehydratase |
| comp27365_c0_seq1 | N-myristoyl transferase |
| comp27593_c0_seq1 | Enoyl-CoA hydratase |
| comp27739_c0_seq2 | PPAP2; phosphatidate phosphatase |
| comp27863_c0_seq1 | Phosphatidylinositol transfer protein |
| comp28291_c0_seq1 | ELOVL4; elongation of very long chain fatty acids protein 4 |
| comp292835_c0_seq1 | Acyl-CoA oxidase |
| comp29355_c0_seq1 | E2.4.1.46; 1,2-diacylglycerol 3-beta-galactosyltransferase |
| comp2953_c0_seq1 | Oxidosqualene-lanosterol cyclase and related proteins |
| comp29767_c0_seq1 | Phosphatidylinositol transfer protein PDR16 and related proteins |
| comp29845_c0_seq1 | 3-hydroxyacyl-CoA dehydrogenase |
| comp300855_c0_seq1 | Long-chain acyl-CoA synthetases (AMP-forming) |
| comp305053_c0_seq1 | Acyl-CoA synthetase |
| comp30763_c0_seq1 | Acyl-CoA synthetase |
| comp31155_c0_seq1 | Squalene synthetase |
| comp31231_c0_seq1 | AGPS; alkyldihydroxyacetonephosphate synthase |
| comp31495_c0_seq1 | STS; steryl-sulfatase |
| comp31660_c0_seq1 | UGT; glucuronosyltransferase |
| comp31772_c0_seq1 | Predicted L-carnitine dehydratase/alpha-methylacyl-CoA racemase |
| comp31802_c0_seq1 | 1-acyl-sn-glycerol-3-phosphate acyltransferase |
| comp32518_c0_seq1 | Peroxisomal long-chain acyl-CoA transporter, ABC superfamily |
| comp32842_c0_seq1 | Enoyl-CoA isomerase |
| comp33276_c0_seq1 | Enoyl-CoA hydratase |
| comp34642_c0_seq1 | Sterol reductase/lamin B receptor |
| comp34716_c0_seq1 | Oxidosqualene-lanosterol cyclase and related proteins |
| comp35922_c0_seq1 | Acyl-CoA-binding protein |
| comp36575_c0_seq1 | Ethanolamine kinase |
| comp36898_c0_seq1 | Phosphatidylserine decarboxylase |
| comp371642_c0_seq1 | Phospholipase D1 |
| comp37430_c0_seq1 | Acyl-CoA synthetase |
| comp37630_c0_seq1 | Enoyl-CoA isomerase |
| comp37678_c0_seq1 | Long-chain acyl-CoA synthetases (AMP-forming) |
| comp37879_c1_seq1 | Fatty acyl-CoA elongase/Polyunsaturated fatty acid specific elongation enzyme |
| comp38079_c0_seq1 | Acetyl-CoA carboxylase |
| comp389844_c0_seq1 | Lysophospholipase |
| comp39020_c0_seq1 | Medium-chain acyl-CoA dehydrogenase |
| comp39257_c0_seq1 | NSDHL, ERG26; sterol-4alpha-carboxylate 3-dehydrogenase (decarboxylating) |
| comp39934_c0_seq1 | Delta 6-fatty acid desaturase/delta-8 sphingolipid desaturase |
| comp40046_c0_seq1 | Predicted phosphate acyltransferase, contains PlsC domain |
| comp40096_c1_seq1 | Enoyl-CoA hydratase/isomerase |
| comp40266_c0_seq3 | fabH; 3-oxoacyl-[acyl-carrier-protein] synthase III |
| comp40294_c0_seq1 | Fatty acyl-CoA elongase/Polyunsaturated fatty acid specific elongation enzyme |
| comp40486_c0_seq1 | 17 beta-hydroxysteroid dehydrogenase type 3, HSD17B3 |
| comp40790_c0_seq1 | accB, bccP; acetyl-CoA carboxylase biotin carboxyl carrier protein |
| comp40975_c0_seq1 | AKR1; aldehyde reductase |
| comp41108_c0_seq1 | LPT1, ALE1; lysophospholipid acyltransferase |
| comp41725_c0_seq1 | HSD11B1; corticosteroid 11-beta-dehydrogenase isozyme 1 |
| comp41814_c0_seq1 | Cis-prenyltransferase |
| comp41827_c0_seq1 | phospholipid:diacylglycerol acyltransferase |
| comp41929_c0_seq1 | Peroxisomal long-chain acyl-CoA transporter, ABC superfamily |
| comp41982_c0_seq1 | Animal-type fatty acid synthase and related proteins |
| comp42212_c0_seq1 | Enoyl-CoA isomerase |
| comp42585_c1_seq1 | CERS; ceramide synthetase |
| comp42861_c0_seq1 | ggt; gamma-glutamyltranspeptidase |
| comp42880_c0_seq1 | Phosphatidylinositol transfer protein PDR16 and related proteins |
| comp43060_c0_seq1 | Acyl-CoA synthetase |
| comp43330_c0_seq1 | microsomal delta-5 desaturase |
| comp43407_c2_seq1 | glutathione peroxidase |
| comp43435_c1_seq2 | Phospholipase D1 |
| comp43480_c0_seq1 | Medium-chain acyl-CoA dehydrogenase |
| comp43673_c0_seq1 | Lysophospholipase |
| comp43696_c0_seq1 | Long chain fatty acid elongase |
| comp44079_c0_seq1 | Enoyl-CoA hydratase |
| comp44082_c0_seq1 | LPCAT1_2; lysophosphatidylcholine acyltransferase / lyso-PAF acetyltransferase |
| comp44349_c0_seq1 | Very long-chain acyl-CoA synthetase/fatty acid transporter |
| comp4466_c0_seq1 | ARSA; arylsulfatase A |
| comp44827_c0_seq1 | Acetyl-CoA carboxylase |
| comp44980_c2_seq1 | CDP-diacylglycerol synthase |
| comp45074_c0_seq1 | Very long-chain acyl-CoA synthetase/fatty acid transporter |
| comp45124_c1_seq1 | Predicted lipase |
| comp45210_c0_seq1 | Fatty acyl-CoA elongase/Polyunsaturated fatty acid specific elongation enzyme |
| comp45318_c0_seq1 | Triglyceride lipase-cholesterol esterase |
| comp45380_c0_seq1 | ASAH2; neutral ceramidase |
| comp45444_c0_seq1 | Short-chain acyl-CoA dehydrogenase |
| comp45685_c0_seq1 | Serine/threonine kinase receptor-associated protein |
| comp45703_c0_seq1 | Very long-chain acyl-CoA synthetase/fatty acid transporter |
| comp45757_c0_seq1 | LYPLA3; lysophospholipase III |
| comp46104_c0_seq1 | Very long-chain acyl-CoA synthetase/fatty acid transporter |
| comp46251_c0_seq1 | fabG; 3-oxoacyl-[acyl-carrier protein] reductase |
| comp46499_c2_seq1 | ACSL, fadD; long-chain acyl-CoA synthetase |
| comp46673_c0_seq1 | PPT; palmitoyl-protein thioesterase |
| comp46879_c0_seq1 | Squalene synthetase |
| comp46897_c0_seq1 | Acyl-CoA synthetase |
| comp46898_c0_seq1 | Sphingosine kinase, involved in sphingolipid metabolism |
| comp47286_c0_seq1 | Malonyl-CoA:ACP transacylase |
| comp47441_c0_seq1 | 3-hydroxyacyl-CoA dehydrogenase |
| comp47540_c0_seq1 | Carnitine O-acyltransferase CPTI |
| comp47628_c0_seq1 | ACADSB; short/branched chain acyl-CoA dehydrogenase |
| comp47628_c0_seq1 | Short-chain acyl-CoA dehydrogenase |
| comp48004_c0_seq1 | Acyl-CoA:diacylglycerol acyltransferase |
| comp48087_c0_seq1 | ASAH2; neutral ceramidase |
| comp48127_c0_seq1 | frmA, ADH5, adhC; S-(hydroxymethyl)glutathione dehydrogenase / alcohol dehydrogenase |
| comp48132_c0_seq1 | Enoyl-CoA hydratase |
| comp48301_c1_seq1 | plcC; phospholipase C |
| comp48599_c0_seq1 | Phospholipase D1 |
| comp48809_c4_seq1 | SQD1, sqdB; UDP-sulfoquinovose synthase |
| comp48881_c0_seq1 | Phosphomannomutase |
| comp49118_c1_seq1 | DAK1, DAK2; dihydroxyacetone kinase |
| comp49278_c2_seq1 | Soluble epoxide hydrolase |
| comp49319_c0_seq1 | Phosphatidylinositol transfer protein |
| comp49324_c0_seq1 | OXCT; 3-oxoacid CoA-transferase |
| comp49392_c1_seq1 | Mitochondrial/plastidial beta-ketoacyl-ACP reductase |
| comp49604_c0_seq1 | Acetyl-CoA carboxylase |
| comp49788_c0_seq1 | Phosphatidylserine decarboxylase |
| comp49900_c0_seq1 | Enoyl-CoA hydratase |
| comp49906_c1_seq1 | Predicted mitochondrial cholesterol transporter |
| comp49931_c0_seq1 | adhE; acetaldehyde dehydrogenase / alcohol dehydrogenase |
| comp50013_c1_seq1 | Predicted lipase |
| comp50257_c0_seq1 | Acetyl-CoA carboxylase |
| comp50320_c0_seq1 | Very long-chain acyl-CoA synthetase/fatty acid transporter |
| comp50381_c2_seq1 | glpK, GK; glycerol kinase |
| comp50419_c0_seq1 | Diacylglycerol kinase |
| comp50499_c1_seq1 | HSD17B1; 17beta-estradiol 17-dehydrogenase |
| comp50582_c1_seq1 | CYP27B; 25-hydroxyvitamin D3 1alpha-hydroxylase |
| comp50696_c4_seq1 | CRLS; cardiolipin synthase |
| comp50789_c0_seq1 | Myo-inositol-1-phosphate synthase |
| comp51059_c1_seq1 | Oxidosqualene-lanosterol cyclase and related proteins |
| comp51070_c0_seq1 | accA; acetyl-CoA carboxylase carboxyl transferase subunit alpha |
| comp51162_c0_seq1 | Predicted phospholipase |
| comp51288_c0_seq3 | serine palmitoyltransferase |
| comp51297_c0_seq1 | Lysophospholipase |
| comp51348_c0_seq1 | 17 beta-hydroxysteroid dehydrogenase type 3, HSD17B3 |
| comp51658_c1_seq1 | Enoyl-CoA hydratase |
| comp51898_c1_seq1 | Phospholipase D1 |
| comp51949_c0_seq1 | Oxidosqualene-lanosterol cyclase and related proteins |
| comp51980_c0_seq1 | Acetyl-CoA acetyltransferase |
| comp52244_c0_seq1 | Mitochondrial/plastidial beta-ketoacyl-ACP reductase |
| comp52340_c0_seq1 | TGL4; TAG lipase / steryl ester hydrolase / phospholipase A2 / LPA acyltransferase |
| comp52447_c0_seq2 | Medium-chain acyl-CoA dehydrogenase |
| comp52591_c1_seq2 | HSD17B1; 17beta-estradiol 17-dehydrogenase |
| comp52647_c0_seq1 | Fatty acid desaturase |
| comp52807_c0_seq1 | sphinganine-1-phosphate aldolase |
| comp53014_c0_seq1 | ALDH7A1; aldehyde dehydrogenase family 7 member A1 |
| comp53126_c0_seq1 | Acetyl-CoA acetyltransferase |
| comp53142_c1_seq1 | Long chain fatty acid elongase |
| comp53150_c0_seq2 | gpsA; glycerol-3-phosphate dehydrogenase (NAD(P)+) |
| comp53252_c0_seq1 | HSD3B; 3beta-hydroxy-delta5-steroid dehydrogenase / steroid delta-isomerase |
| comp53254_c0_seq1 | 3-oxoacyl CoA thiolase |
| comp53269_c0_seq1 | PTGES2; microsomal prostaglandin-E synthase 2 |
| comp53298_c2_seq4 | Myo-inositol-1-phosphate synthase |
| comp53361_c0_seq1 | linoleoyl-CoA desaturase |
| comp53386_c0_seq1 | 2-enoyl-CoA hydratase/3-hydroxyacyl-CoA dehydrogenase/Peroxisomal 3-ketoacyl-CoA-thiolase, sterol-binding domain and related enzymes |
| comp53531_c0_seq1 | 3-hydroxyacyl-CoA dehydrogenase |
| comp53629_c0_seq1 | N-myristoyl transferase |
| comp53645_c0_seq1 | Acetyl-CoA acetyltransferase |
| comp53720_c0_seq1 | Oxidosqualene-lanosterol cyclase and related proteins |
| comp53772_c0_seq1 | Sterol C5 desaturase |
| comp54120_c0_seq1 | Acetyl-CoA acetyltransferase |
| comp54157_c1_seq1 | fabK; enoyl-[acyl-carrier protein] reductase II |
| comp54188_c0_seq1 | Hydroxyacyl-CoA dehydrogenase/enoyl-CoA hydratase |
| comp54221_c1_seq1 | galA, rafA; alpha-galactosidase |
| comp54286_c0_seq2 | Hormone-sensitive lipase HSL |
| comp54291_c0_seq1 | Long-chain acyl-CoA synthetases (AMP-forming) |
| comp54298_c2_seq1 | AGPAT3_4; lysophosphatidic acid acyltransferase / lysophosphatidylinositol acyltransferase |
| comp54318_c0_seq1 | Fatty acid desaturase |
| comp54322_c1_seq1 | Medium-chain acyl-CoA dehydrogenase |
| comp54429_c0_seq1 | PHS1, PAS2; very-long-chain (3R)-3-hydroxyacyl-[acyl-carrier protein] dehydratase |
| comp54631_c10_seq1 | CDS1, CDS2, cdsA; phosphatidate cytidylyltransferase |
| comp54685_c1_seq1 | Peroxisomal long-chain acyl-CoA transporter, ABC superfamily |
| comp54695_c3_seq1 | frmA, ADH5, adhC; S-(hydroxymethyl)glutathione dehydrogenase / alcohol dehydrogenase |
| comp54712_c0_seq1 | Acetyl-CoA acetyltransferase |
| comp54849_c0_seq1 | glutathione peroxidase |
| comp54888_c0_seq1 | HADH; 3-hydroxyacyl-CoA dehydrogenase |
| comp54929_c0_seq1 | glutathione peroxidase |
| comp54958_c0_seq1 | Hydroxymethylglutaryl-CoA synthase |
| comp55039_c1_seq1 | CHO1, pssA; CDP-diacylglycerol---serine O-phosphatidyltransferase |
| comp55040_c0_seq1 | GCDH, gcdH; glutaryl-CoA dehydrogenase |
| comp55055_c12_seq9 | SQD2; sulfoquinovosyltransferase |
| comp55079_c0_seq1 | ACSBG; long-chain-fatty-acid--CoA ligase ACSBG |
| comp55177_c0_seq1 | ALOX12B; arachidonate 12-lipoxygenase (R-type) |
| comp55187_c0_seq1 | Short-chain acyl-CoA dehydrogenase |
| comp55216_c0_seq1 | Enoyl-CoA hydratase |
| comp55285_c4_seq2 | 3-oxoacyl CoA thiolase |
| comp55444_c0_seq1 | delta5 fatty acid desaturase |
| comp55479_c2_seq2 | aldehyde dehydrogenase (NAD+) |
| comp55500_c0_seq1 | Myo-inositol-1-phosphate synthase |
| comp55513_c0_seq2 | 3-hydroxy-3-methylglutaryl-CoA (HMG-CoA) reductase |
| comp55552_c3_seq1 | Acyl-CoA synthetase |
| comp55567_c1_seq1 | Putative phosphoinositide phosphatase |
| comp55659_c1_seq1 | Acetyl-CoA carboxylase |
| comp55686_c8_seq1 | digalactosyldiacylglycerol synthase |
| comp55703_c0_seq1 | HMGCL, hmgL; hydroxymethylglutaryl-CoA lyase |
| comp55774_c0_seq1 | Acetyl-CoA carboxylase |
| comp55776_c16_seq1 | Long-chain acyl-CoA synthetases (AMP-forming) |
| comp55788_c0_seq1 | Predicted phosphate acyltransferase, contains PlsC domain |
| comp55854_c0_seq1 | fatty acid desaturase domain protein |
| comp55967_c8_seq3 | PLD1_2; phospholipase D1/2 |
| comp55997_c0_seq4 | Acyl-CoA synthetase |
| comp56096_c19_seq1 | SQLE, ERG1; squalene monooxygenase |
| comp56138_c0_seq1 | Squalene synthetase |
| comp56195_c0_seq1 | aldehyde dehydrogenase (NAD+) |
| comp56272_c1_seq1 | Peroxisomal long-chain acyl-CoA transporter, ABC superfamily |
| comp56283_c0_seq1 | Phosphatidylinositol transfer protein SEC14 and related proteins |
| comp56318_c9_seq2 | ACOX1, ACOX3; acyl-CoA oxidase |
| comp56349_c0_seq1 | Myo-inositol-1-phosphate synthase |
| comp5642_c0_seq1 | sn-1,2-diacylglycerol ethanolamine- and cholinephosphotranferases |
| comp56425_c4_seq1 | Choline phosphate cytidylyltransferase/Predicted CDP-ethanolamine synthase |
| comp56447_c0_seq1 | CYP51; sterol 14-demethylase |
| comp56619_c0_seq1 | accC; acetyl-CoA carboxylase, biotin carboxylase subunit |
| comp56708_c0_seq1 | Fatty acid desaturase |
| comp56753_c0_seq1 | ELOVL2; elongation of very long chain fatty acids protein 2 |
| comp56754_c0_seq1 | LTA4H; leukotriene-A4 hydrolase |
| comp56818_c0_seq1 | Delta 6-fatty acid desaturase/delta-8 sphingolipid desaturase |
| comp56840_c0_seq1 | fabI; enoyl-[acyl-carrier protein] reductase I |
| comp56869_c0_seq1 | fabF; 3-oxoacyl-[acyl-carrier-protein] synthase II |
| comp56906_c0_seq1 | E5.3.99.2, PTGDS; prostaglandin-H2 D-isomerase |
| comp57028_c0_seq1 | Long-chain acyl-CoA synthetases (AMP-forming) |
| comp57069_c0_seq1 | MECR, NRBF1; mitochondrial trans-2-enoyl-CoA reductase |
| comp57087_c0_seq1 | galA, rafA; alpha-galactosidase |
| comp57274_c0_seq1 | ACSBG; long-chain-fatty-acid--CoA ligase ACSBG |
| comp57481_c0_seq1 | DHCR24; delta24-sterol reductase |
| comp57492_c0_seq1 | DOX1; alpha-dioxygenase |
| comp57673_c0_seq1 | CBR3; carbonyl reductase 3 |
| comp57785_c0_seq1 | Phospholipase A2-activating protein (contains WD40 repeats) |
| comp57946_c0_seq1 | ATS1; glycerol-3-phosphate O-acyltransferase |
| comp57996_c0_seq1 | Serine/threonine kinase receptor-associated protein |
| comp58235_c0_seq1 | plsC; 1-acyl-sn-glycerol-3-phosphate acyltransferase |
| comp58324_c0_seq1 | Myo-inositol-1-phosphate synthase |
| comp60191_c0_seq1 | Acyl-CoA-binding protein |
| comp62133_c0_seq1 | Acetyl-CoA acetyltransferase |
| comp62860_c0_seq1 | accA; acetyl-CoA carboxylase carboxyl transferase subunit alpha |
| comp67095_c0_seq1 | Protein involved in plasmid maintenance/nuclear protein involved in lipid metabolism |
| comp71267_c0_seq1 | PTGDS; prostaglandin-H2 D-isomerase |
| comp71341_c0_seq1 | ggt; gamma-glutamyltranspeptidase |
| comp83229_c0_seq1 | SMT1, ERG6; sterol 24-C-methyltransferase |
| comp8600_c0_seq1 | C-4 sterol methyl oxidase |
| comp90337_c0_seq1 | Hydroxymethylglutaryl-CoA synthase |

The genes marked with yellow color are analyzed in our manuscript.
